# Supplementary material for: Structure sensitivity of Cu and CuZn catalysts relevant to industrial methanol synthesis
Source: Nat Commun. 2016 Oct 5;7:13057. doi: 10.1038/ncomms13057 (PMC5476790; doi:10.1038/ncomms13057)
Supplement: Supplementary Information — Supplementary Figures 1-18, Supplementary Tables 1-6, Supplementary Methods and Supplementary References [file ncomms13057-s1.pdf]

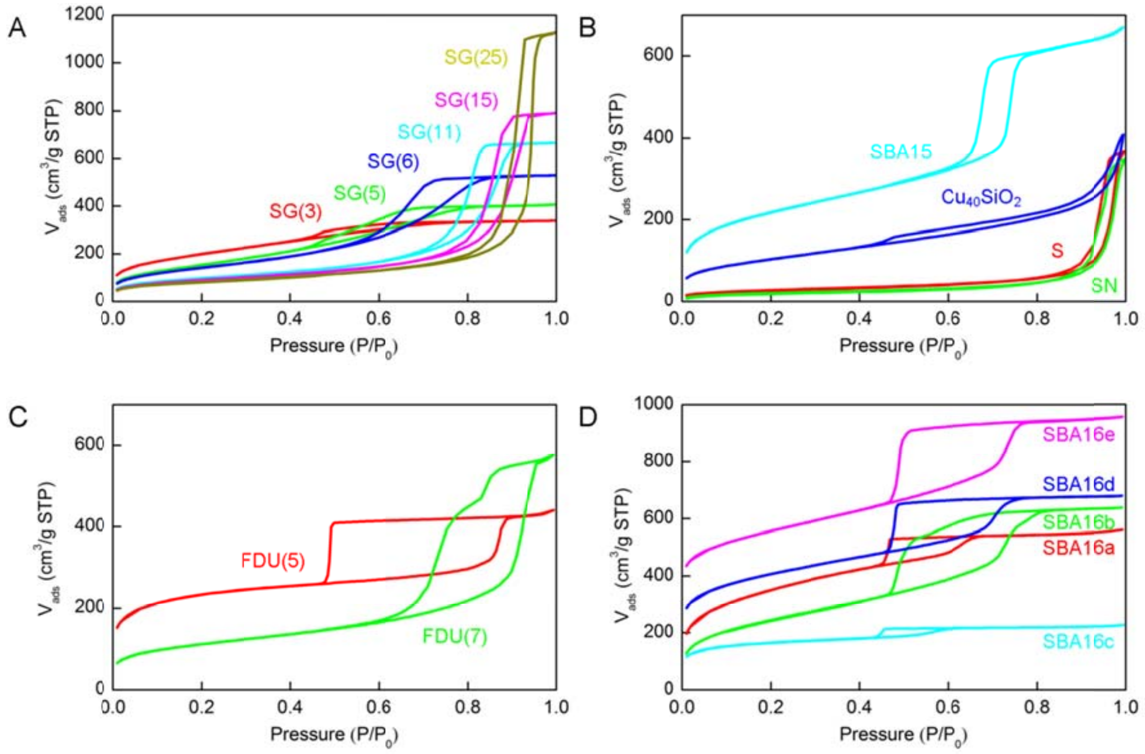

Supplementary Figure 1

N_2-physisorption isotherms of the silica supports. (A) six different silica gels, (B) Stöber silica (S), functionalized Stöber silica (SN), Cu/SiO_2 prepared via precipitation followed by reduction and SBA15, (C) two FDU supports and (D) SBA16 supports.

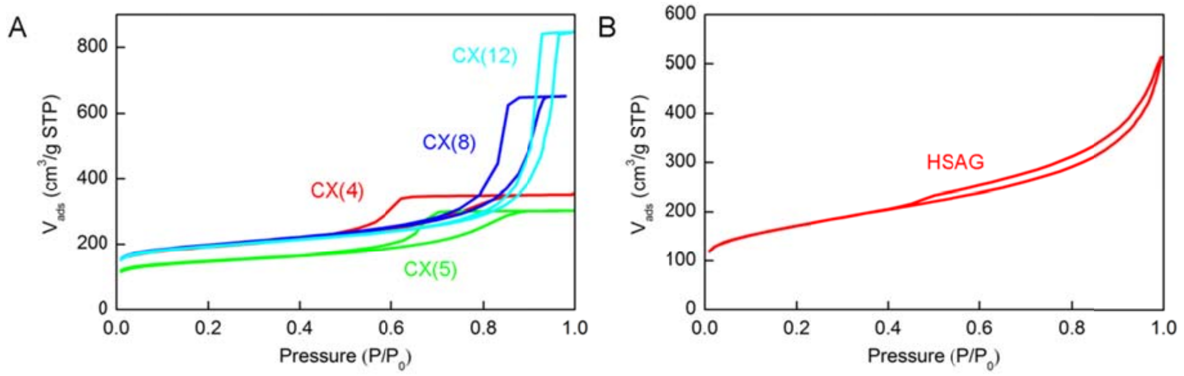

Supplementary Figure 2

N_2-physisorption isotherms of the carbon supports. (A) four different carbon xerogels and (B) high surface area graphite.

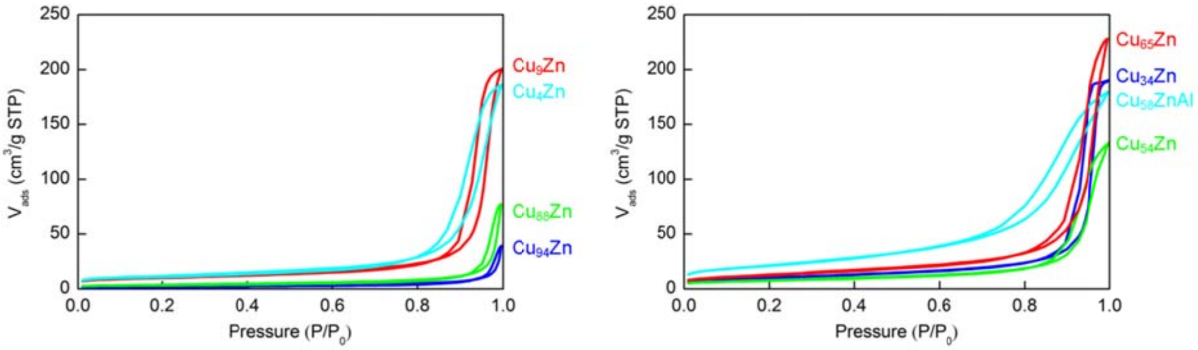

Supplementary Figure 3

N_2-physisorption isotherms of the precipitated Cu/ZnO(/Al_2O_3) samples after reduction and passivation.

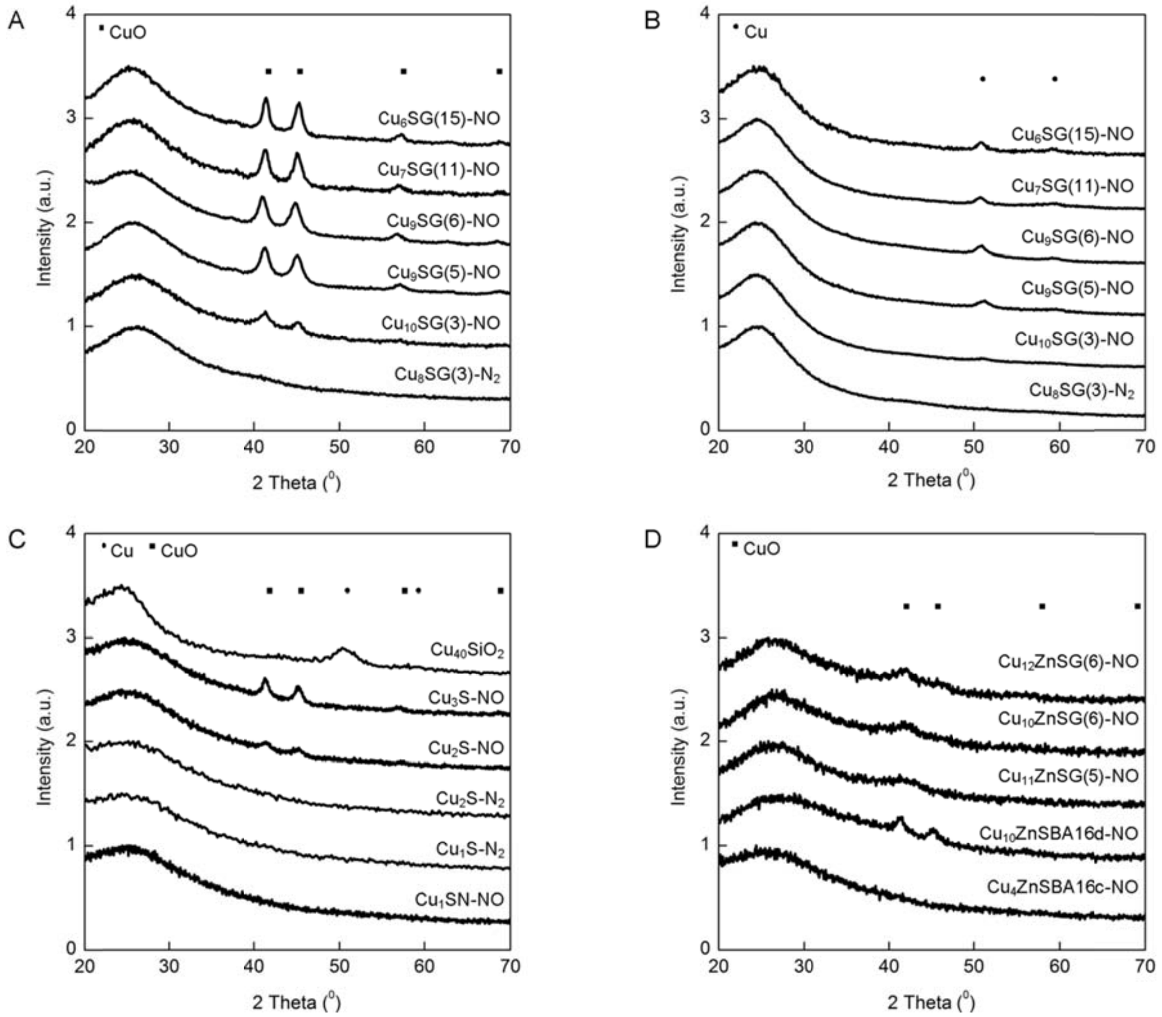
A
CuO
$Cu_6SG(15)$-NO
$Cu_7SG(11)$-NO
$Cu_9SG(6)$-NO
$Cu_9SG(5)$-NO
$Cu_{10}SG(3)$-NO
$Cu_8SG(3)$-N_2
Intensity (a.u.)
2 Theta (^{0})
B
Cu
$Cu_6SG(15)$-NO
$Cu_7SG(11)$-NO
$Cu_9SG(6)$-NO
$Cu_9SG(5)$-NO
$Cu_{10}SG(3)$-NO
$Cu_8SG(3)$-N_2
Intensity (a.u.)
2 Theta (^{0})
C
Cu
CuO
$Cu_{40}SiO_2$
Cu_3S-NO
Cu_2S-NO
Cu_2S-N_2
Cu_1S-N_2
Cu_1SN-NO
Intensity (a.u.)
2 Theta (^{0})
D
CuO
$Cu_{12}ZnSG(6)$-NO
$Cu_{10}ZnSG(6)$-NO
$Cu_{11}ZnSG(5)$-NO
$Cu_{10}ZnSBA16d$-NO
$Cu_4ZnSBA16c$-NO
Intensity (a.u.)
2 Theta (^{0})

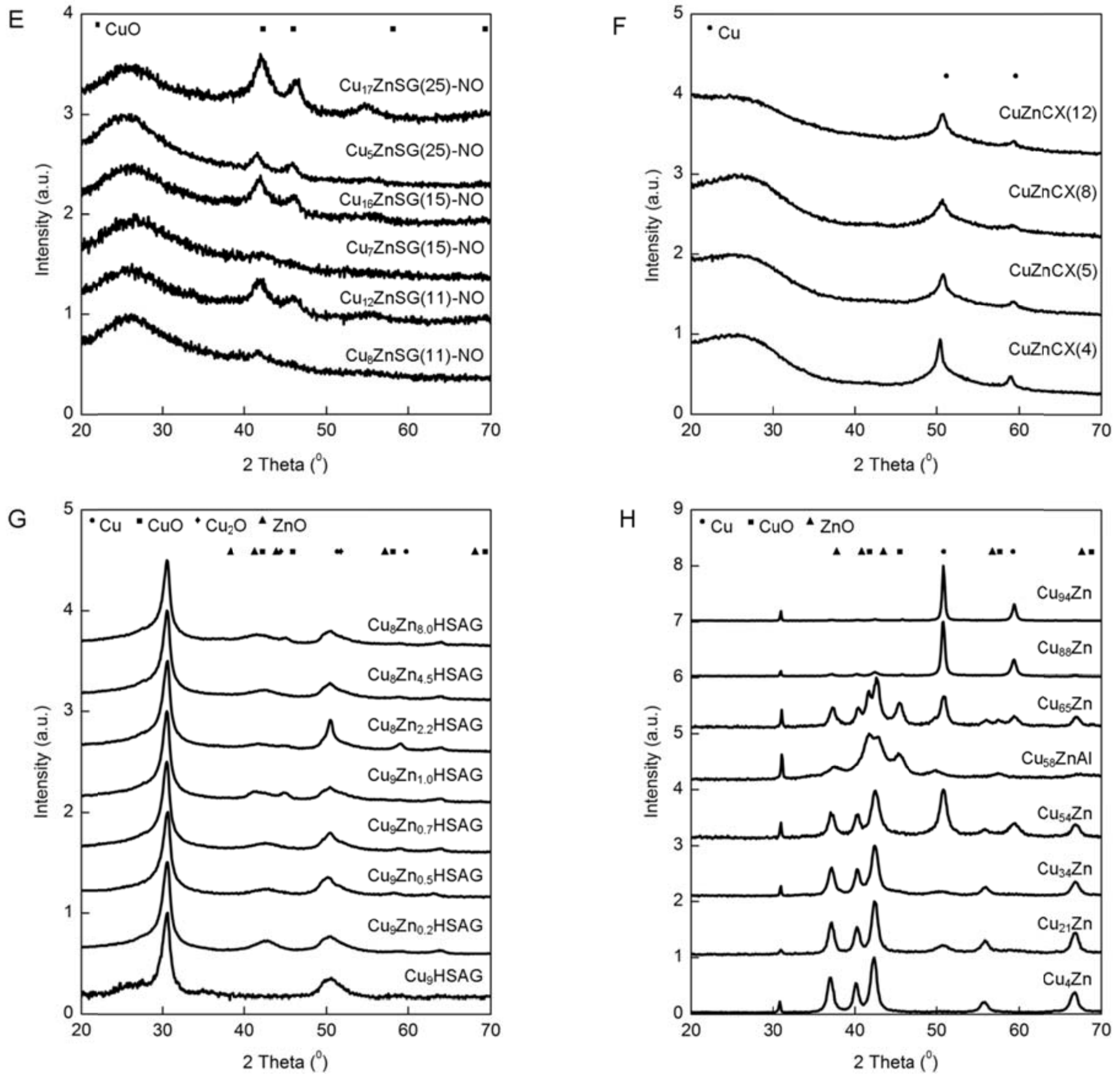

Supplementary Figure 4

X-ray diffractograms of the different samples. (A) Cu/silica gel after heat treatment in N_2 or 2% NO/N_2, (B) Cu/silica gel after reduction and passivation, (C) Cu/SiO_2, (D and E) $CuZn/SiO_2$, (F) CuZn/CX, (G) CuZn/HSAG and (H) $Cu/ZnO(/Al_2O_3)$. For Cu(Zn) on carbon or on silica the spectra have been normalized to the peak corresponding to the support. For $Cu/ZnO(/Al_2O_3)$ the spectra are normalized on the highest peak. The small peaks at 31° 2 theta in image H are due to the graphite lubricant used for pelletization.

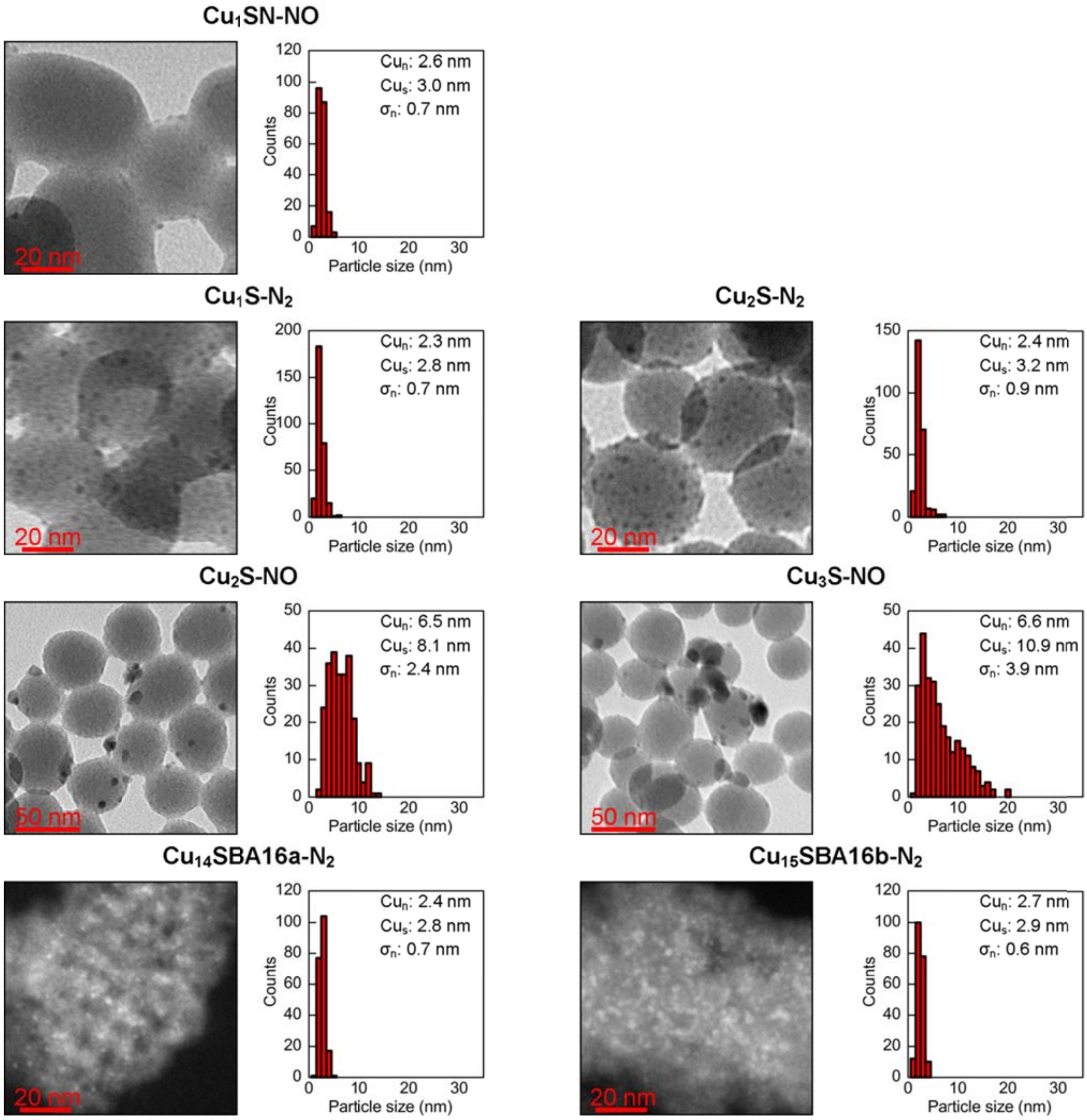
Cu1SN-NO
Cun: 2.6 nm
Cus: 3.0 nm
σn: 0.7 nm
Cu1S-N2
Cun: 2.3 nm
Cus: 2.8 nm
σn: 0.7 nm
Cu2S-N2
Cun: 2.4 nm
Cus: 3.2 nm
σn: 0.9 nm
Cu2S-NO
Cun: 6.5 nm
Cus: 8.1 nm
σn: 2.4 nm
Cu3S-NO
Cun: 6.6 nm
Cus: 10.9 nm
σn: 3.9 nm
Cu14SBA16a-N2
Cun: 2.4 nm
Cus: 2.8 nm
σn: 0.7 nm
Cu15SBA16b-N2
Cun: 2.7 nm
Cus: 2.9 nm
σn: 0.6 nm
20 nm
50 nm
Counts
Particle size (nm)

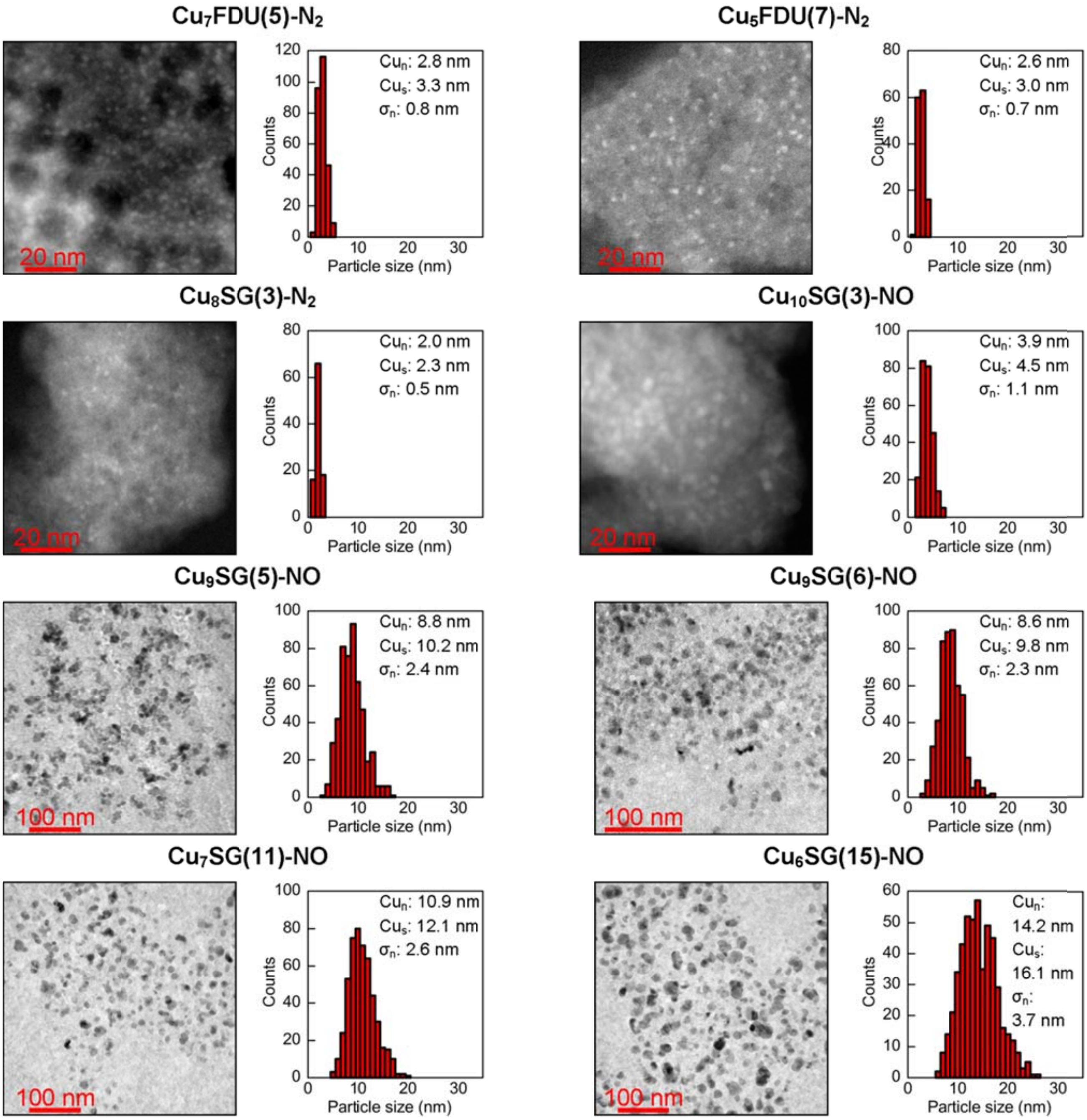

$Cu_7FDU(5)$-N_2
20 nm
Cu_n: 2.8 nm
Cu_s: 3.3 nm
σ_n: 0.8 nm
Counts
Particle size (nm)
$Cu_5FDU(7)$-N_2
20 nm
Cu_n: 2.6 nm
Cu_s: 3.0 nm
σ_n: 0.7 nm
$Cu_8SG(3)$-N_2
20 nm
Cu_n: 2.0 nm
Cu_s: 2.3 nm
σ_n: 0.5 nm
$Cu_{10}SG(3)$-NO
20 nm
Cu_n: 3.9 nm
Cu_s: 4.5 nm
σ_n: 1.1 nm
$Cu_9SG(5)$-NO
100 nm
Cu_n: 8.8 nm
Cu_s: 10.2 nm
σ_n: 2.4 nm
$Cu_9SG(6)$-NO
100 nm
Cu_n: 8.6 nm
Cu_s: 9.8 nm
σ_n: 2.3 nm
$Cu_7SG(11)$-NO
100 nm
Cu_n: 10.9 nm
Cu_s: 12.1 nm
σ_n: 2.6 nm
$Cu_6SG(15)$-NO
100 nm
Cu_n: 14.2 nm
Cu_s: 16.1 nm
σ_n: 3.7 nm

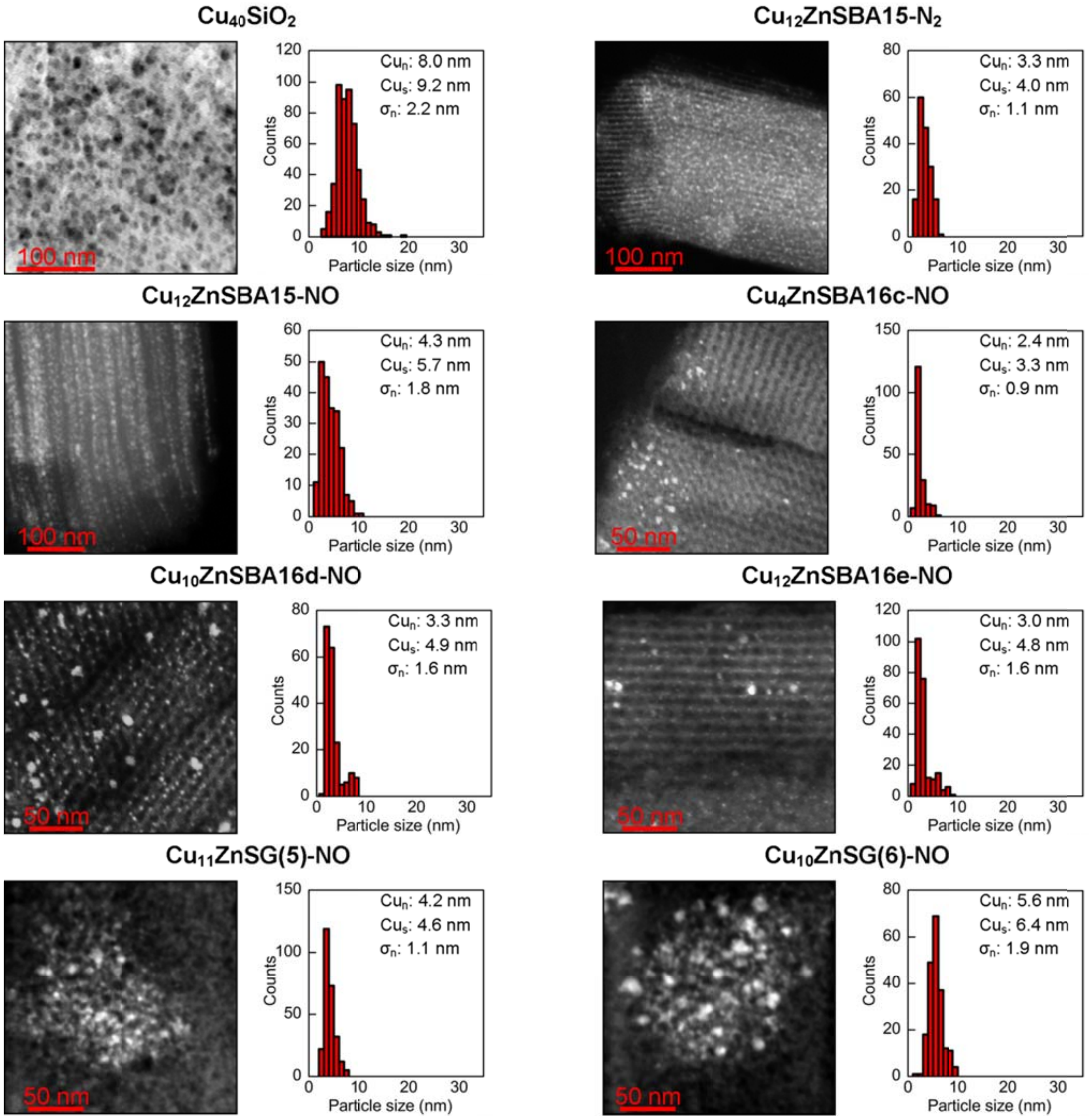

$Cu_{40}SiO_2$
100 nm
Cu_n: 8.0 nm
Cu_s: 9.2 nm
σ_n: 2.2 nm
Counts
Particle size (nm)
$Cu_{12}ZnSBA15-N_2$
100 nm
Cu_n: 3.3 nm
Cu_s: 4.0 nm
σ_n: 1.1 nm
$Cu_{12}ZnSBA15$-NO
100 nm
Cu_n: 4.3 nm
Cu_s: 5.7 nm
σ_n: 1.8 nm
$Cu_4ZnSBA16c$-NO
50 nm
Cu_n: 2.4 nm
Cu_s: 3.3 nm
σ_n: 0.9 nm
$Cu_{10}ZnSBA16d$-NO
50 nm
Cu_n: 3.3 nm
Cu_s: 4.9 nm
σ_n: 1.6 nm
$Cu_{12}ZnSBA16e$-NO
50 nm
Cu_n: 3.0 nm
Cu_s: 4.8 nm
σ_n: 1.6 nm
$Cu_{11}ZnSG(5)$-NO
50 nm
Cu_n: 4.2 nm
Cu_s: 4.6 nm
σ_n: 1.1 nm
$Cu_{10}ZnSG(6)$-NO
50 nm
Cu_n: 5.6 nm
Cu_s: 6.4 nm
σ_n: 1.9 nm

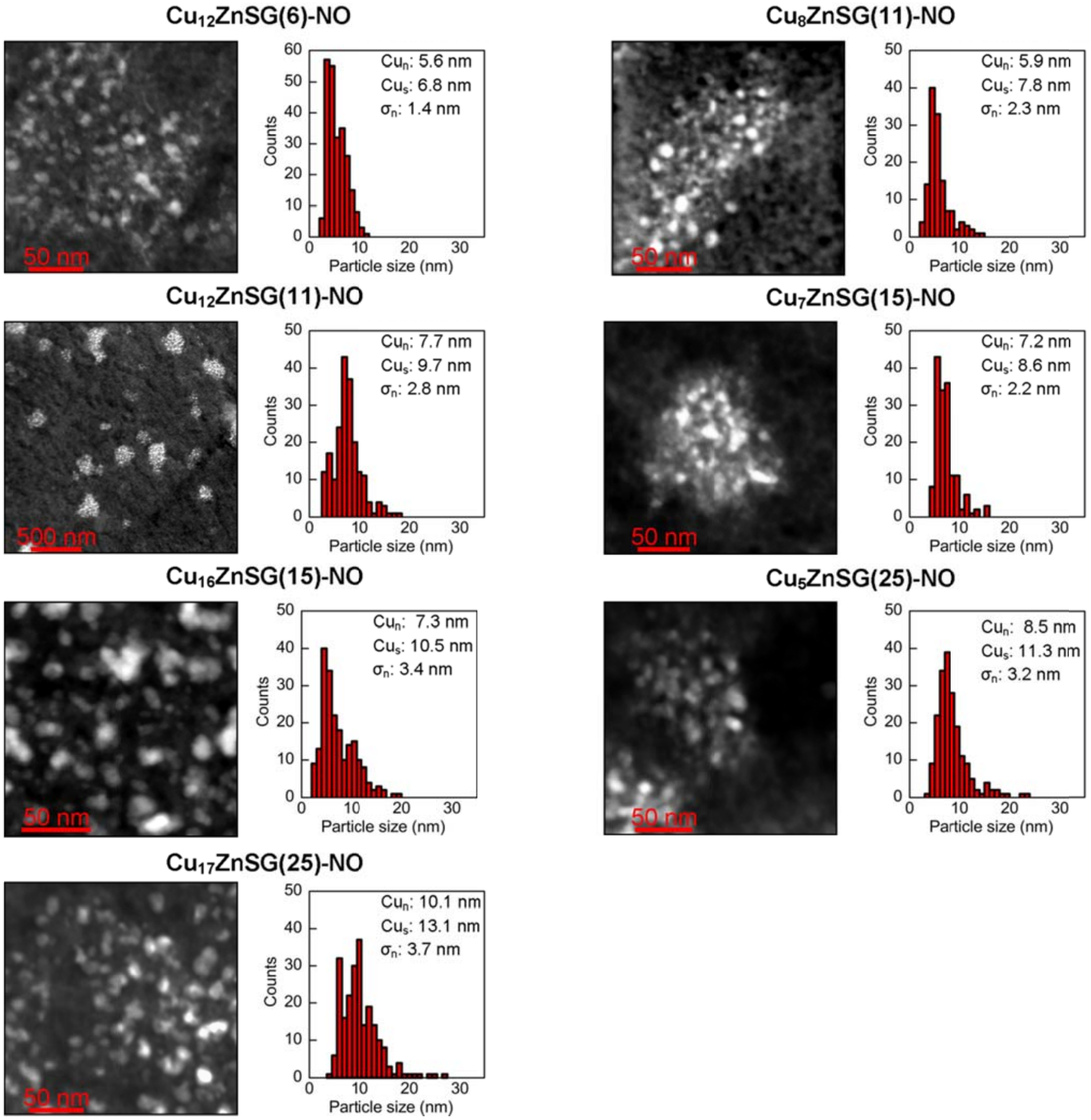

Supplementary Figure 5

(S)TEM images of Cu(Zn)/SiO_2 samples and corresponding particle size distributions, number-averaged particle size (Cu_n), surface-averaged particle size (Cu_s), and standard deviation of the particle size distribution (σ_n).

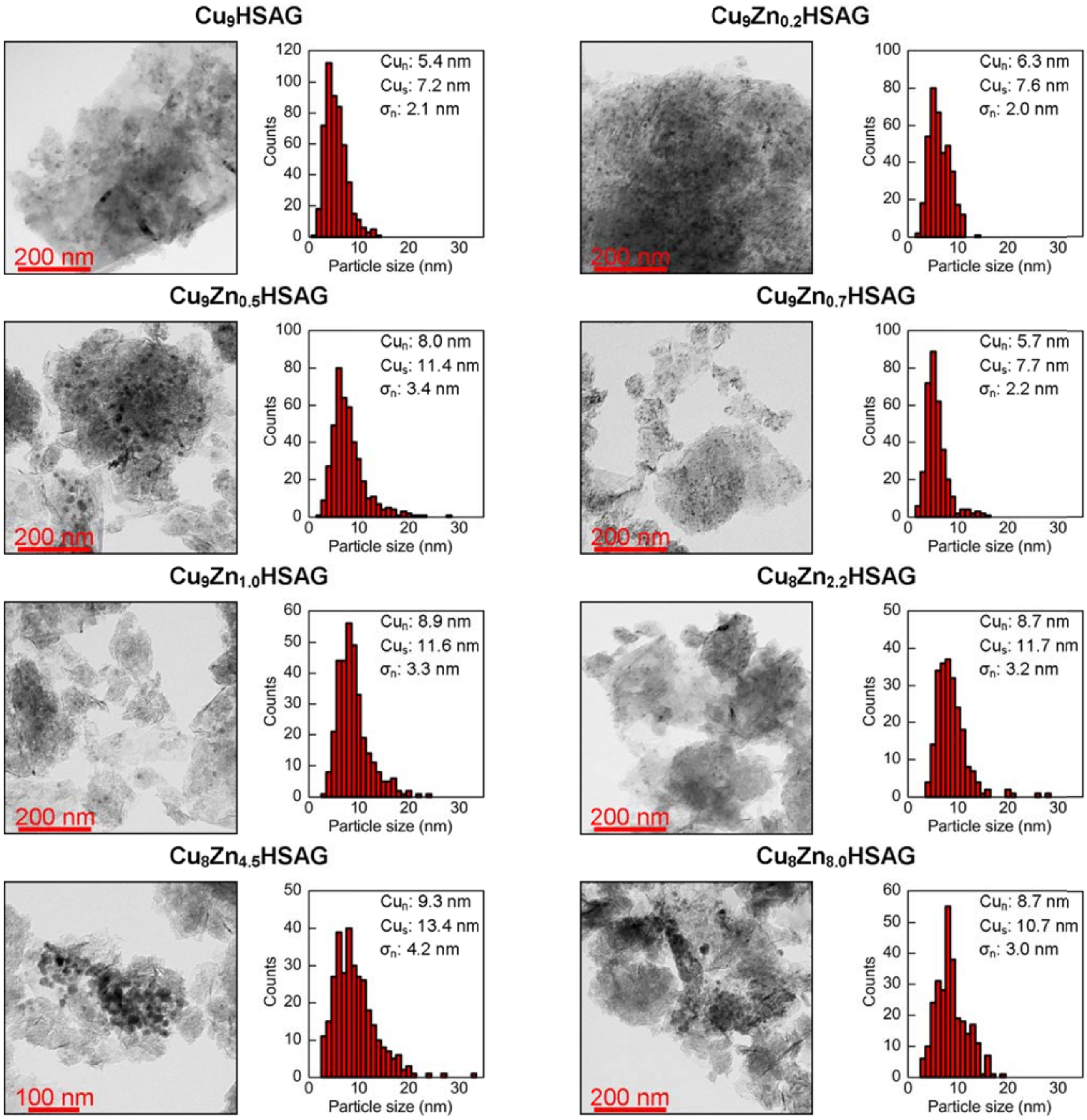
Cu_9HSAG
200 nm
Cu_n: 5.4 nm
Cu_s: 7.2 nm
σ_n: 2.1 nm
$Cu_9Zn_{0.2}HSAG$
200 nm
Cu_n: 6.3 nm
Cu_s: 7.6 nm
σ_n: 2.0 nm
$Cu_9Zn_{0.5}HSAG$
200 nm
Cu_n: 8.0 nm
Cu_s: 11.4 nm
σ_n: 3.4 nm
$Cu_9Zn_{0.7}HSAG$
200 nm
Cu_n: 5.7 nm
Cu_s: 7.7 nm
σ_n: 2.2 nm
$Cu_9Zn_{1.0}HSAG$
200 nm
Cu_n: 8.9 nm
Cu_s: 11.6 nm
σ_n: 3.3 nm
$Cu_8Zn_{2.2}HSAG$
200 nm
Cu_n: 8.7 nm
Cu_s: 11.7 nm
σ_n: 3.2 nm
$Cu_8Zn_{4.5}HSAG$
100 nm
Cu_n: 9.3 nm
Cu_s: 13.4 nm
σ_n: 4.2 nm
$Cu_8Zn_{8.0}HSAG$
200 nm
Cu_n: 8.7 nm
Cu_s: 10.7 nm
σ_n: 3.0 nm
Counts
Particle size (nm)

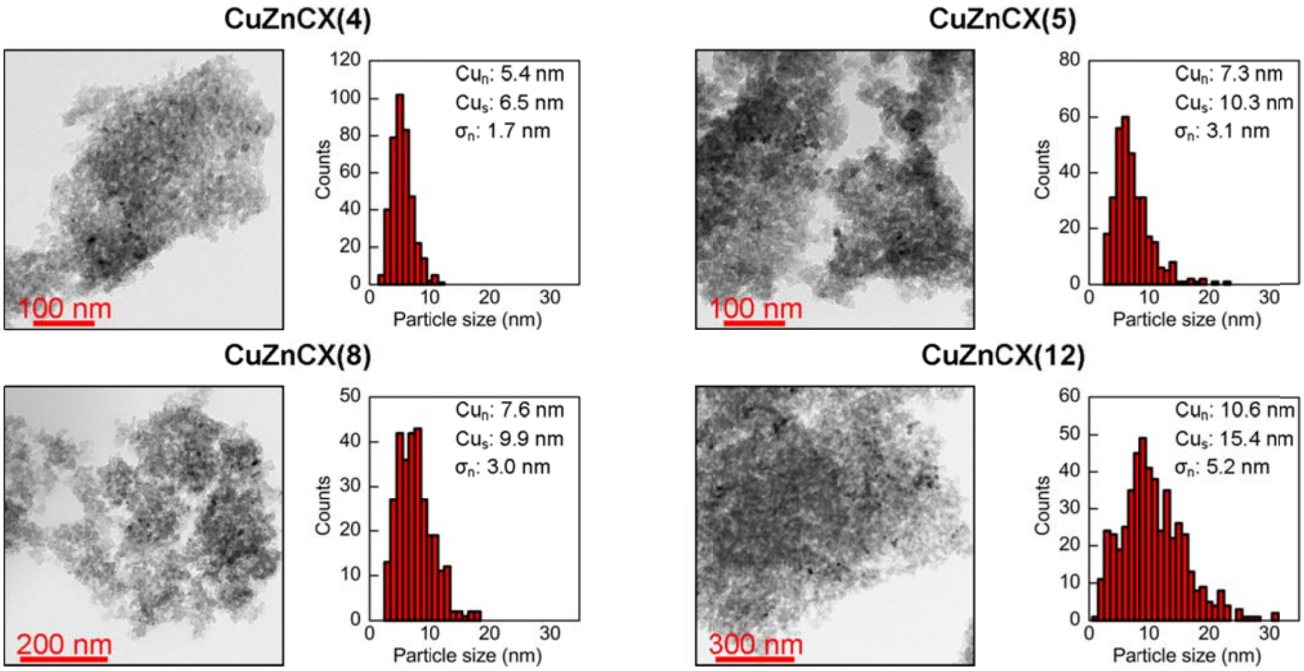

Supplementary Figure 6

TEM images of Cu(Zn)/C samples and corresponding particle size distributions, number-averaged particle size (Cu_n), surface-averaged particle size (Cu_s), and standard deviation of the particle size distribution (σ_n).

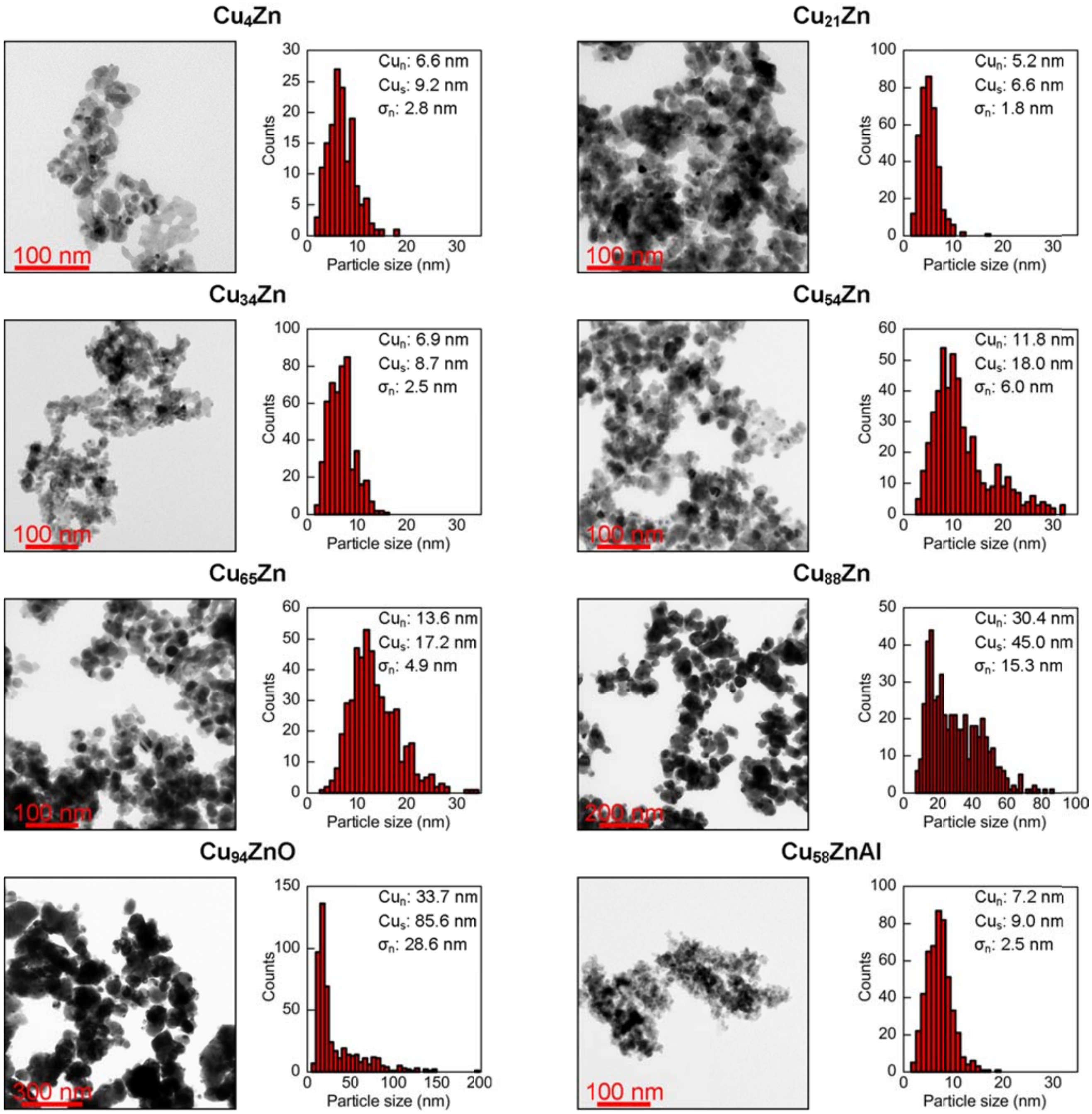

Supplementary Figure 7

TEM images of Cu/ZnO(/Al_2O_3) samples and corresponding particle size distributions, number-averaged particle size (Cu_n), surface-averaged particle size (Cu_s), and standard deviation of the particle size distribution (σ_n).

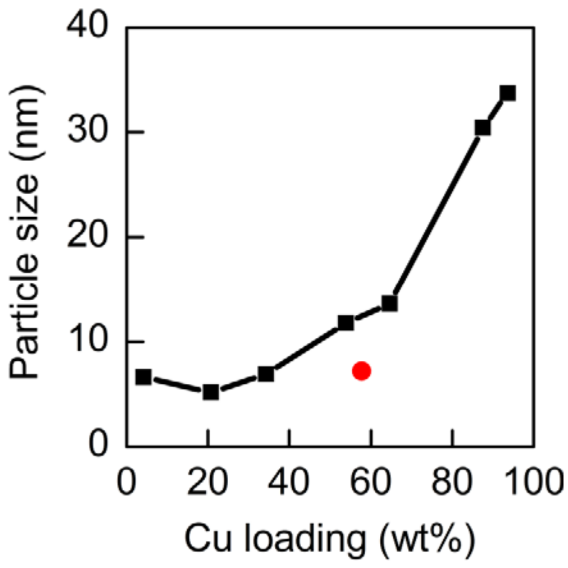

Supplementary Figure 8

TEM number-averaged copper particle size as a function of the copper loading for Cu/ZnO (black) and Cu/ZnO/Al_2O_3 (red). Cu/ZnO/Al_2O_3 had a lower copper particle size than Cu/ZnO with comparable copper loadings.

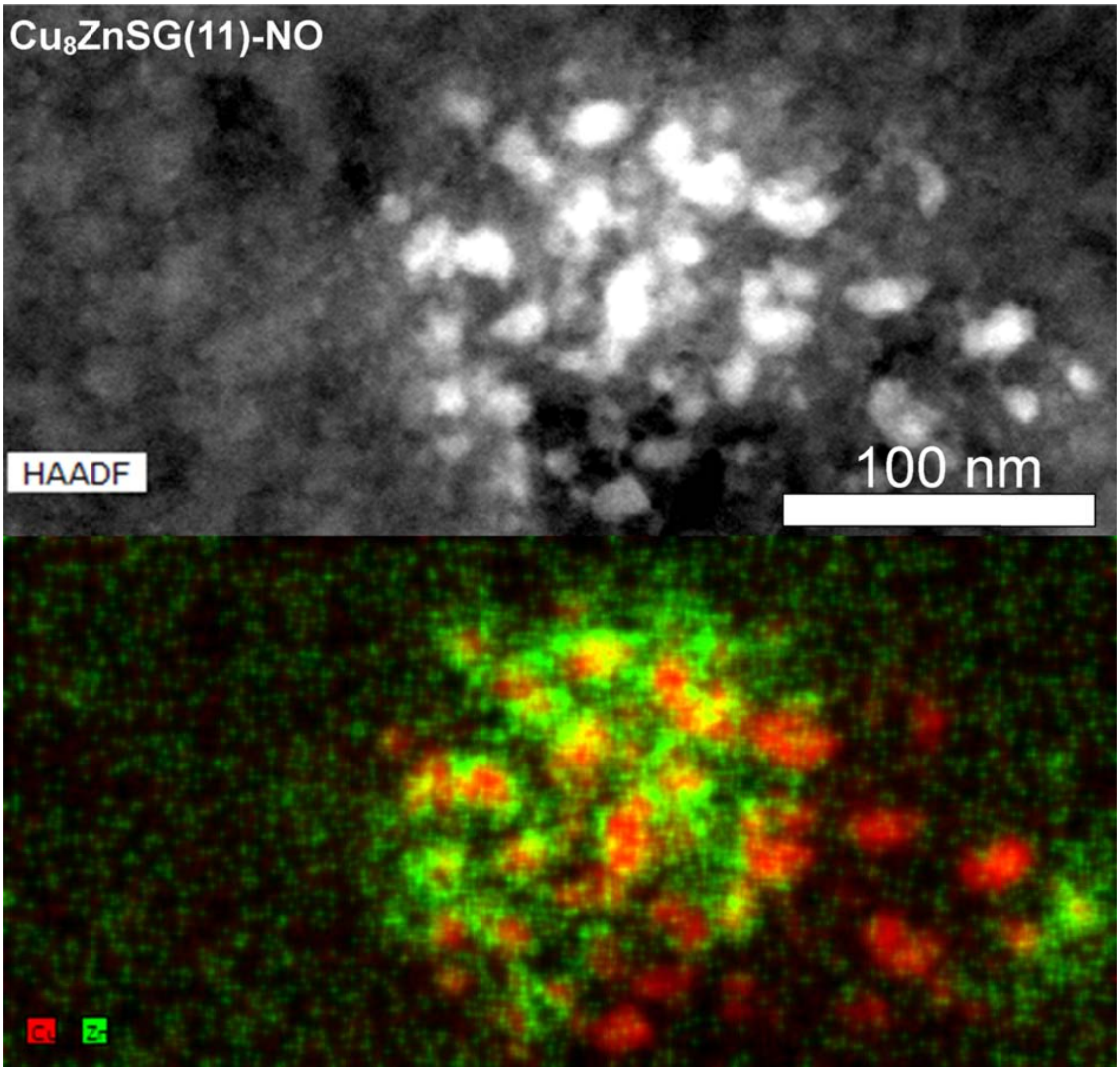

Supplementary Figure 9

STEM-HAADF image and corresponding EDX map with a pixel size of 0.62 nm of a microtomed section of $Cu_8ZnSG(11)$-NO using the TALOS F200x microscope. EDX spectra were processed using the Esprit software (Bruker AXS). Color intensities correspond to the integrated intensities of the Cu-K and Zn-K signal after background correction and are normalized per element. A smooth (3) map filter is used. The average Cu:Zn atomic ratio in the region is 90:10, as determined by using a k-factor of 1.820 and 1.948 for Cu and Zn, respectively. The average size of the copper containing particles is 7.4 ± 1.9 nm (25 particles counted).

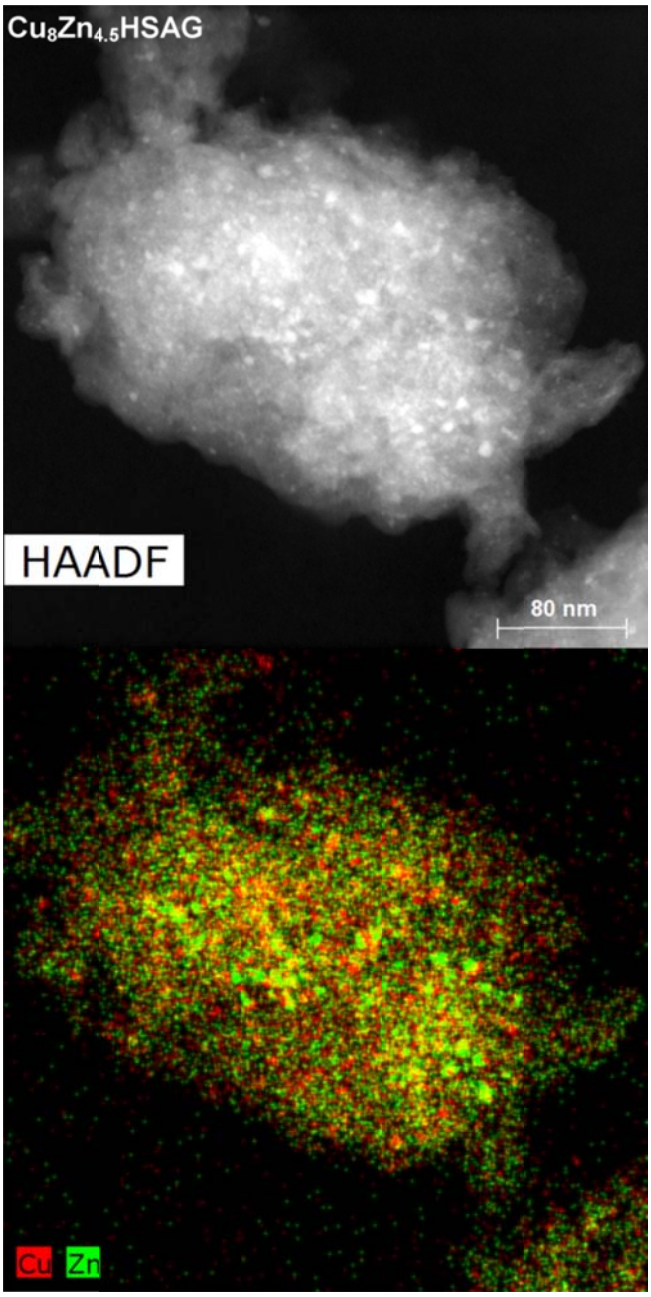

Supplementary Figure 10

STEM-HAADF image and corresponding EDX map with a pixel size of 0.51 nm of $Cu_8Zn_{4.5}$HSAG using the TALOS F200x microscope. EDX spectra were processed using the Esprit software (Bruker AXS). Color intensities correspond to the integrated intensities of the Cu-K and Zn-K signal after background correction and are normalized per element. The average Cu:Zn atomic ratio in the region is 63:37, as determined by using a k-factor of 1.820 and 1.948 for Cu and Zn, respectively. The average size of the copper containing particles is 7.4 ± 2.1 nm (25 particles counted).

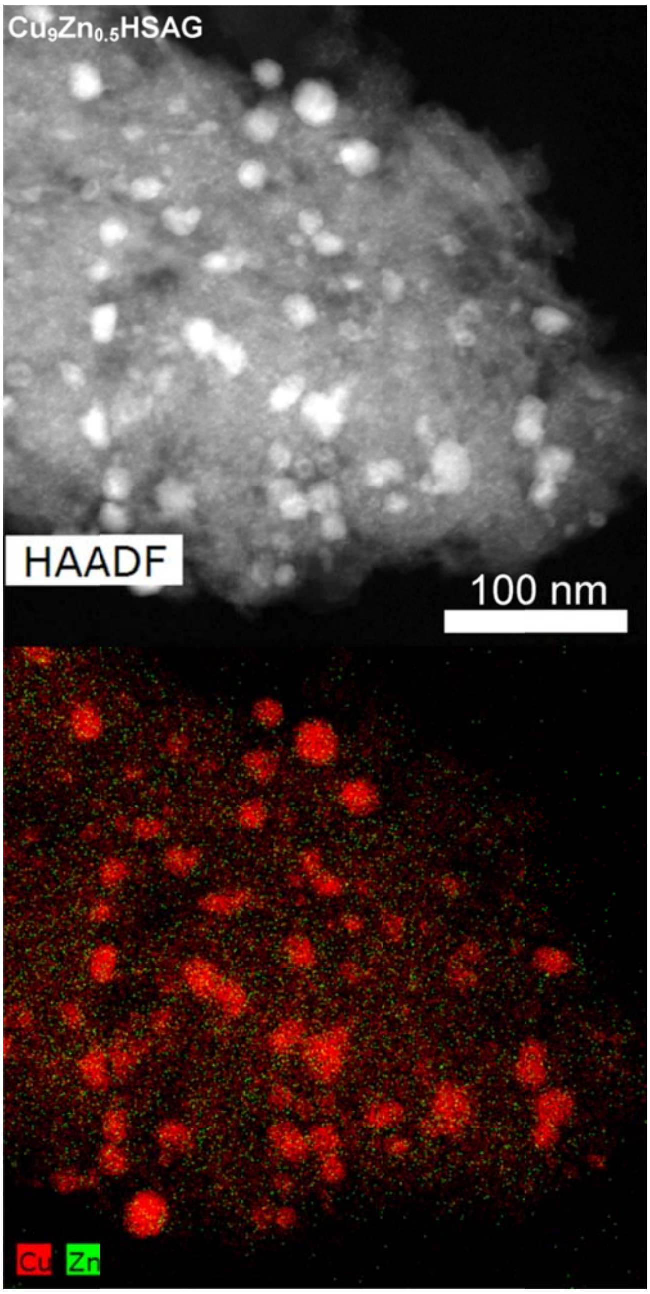

Supplementary Figure 11

STEM-HAADF image and corresponding EDX map with a pixel size of 0.68 nm of $Cu_9Zn_{0.5}$HSAG using the TALOS F200x microscope. EDX spectra were processed using the Esprit software (Bruker AXS). Color intensities correspond to the integrated intensities of the Cu-K and Zn-K signal after background correction and are normalized per element. The average Cu:Zn atomic ratio in the region is 96:4, as determined by using a k-factor of 1.820 and 1.948 for Cu and Zn, respectively. The average size of the copper containing particles is 9.5 ± 3.2 nm (25 particles counted).

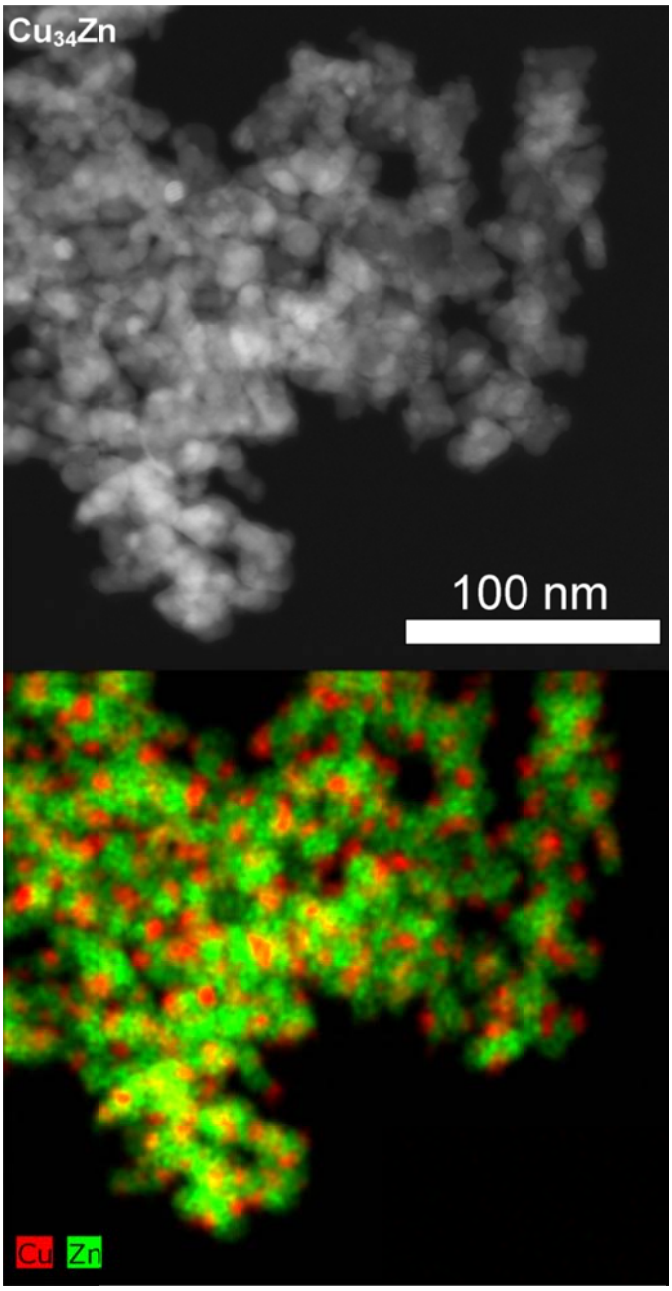

Supplementary Figure 12

STEM-HAADF image and corresponding EDX map with a pixel size of 0.24 nm of $Cu_{34}Zn$ using the TALOS F200x microscope. EDX spectra were processed using the Esprit software (Bruker AXS). Color intensities correspond to the integrated intensities of the Cu-K and Zn-K signal after background correction and are normalized per element. An automatic map filter is used. The average Cu:Zn atomic ratio in the region is 35:65, as determined by using a k-factor of 1.820 and 1.948 for Cu and Zn, respectively. The average size of the copper containing particles is 6.6 ± 1.3 nm (25 particles counted).

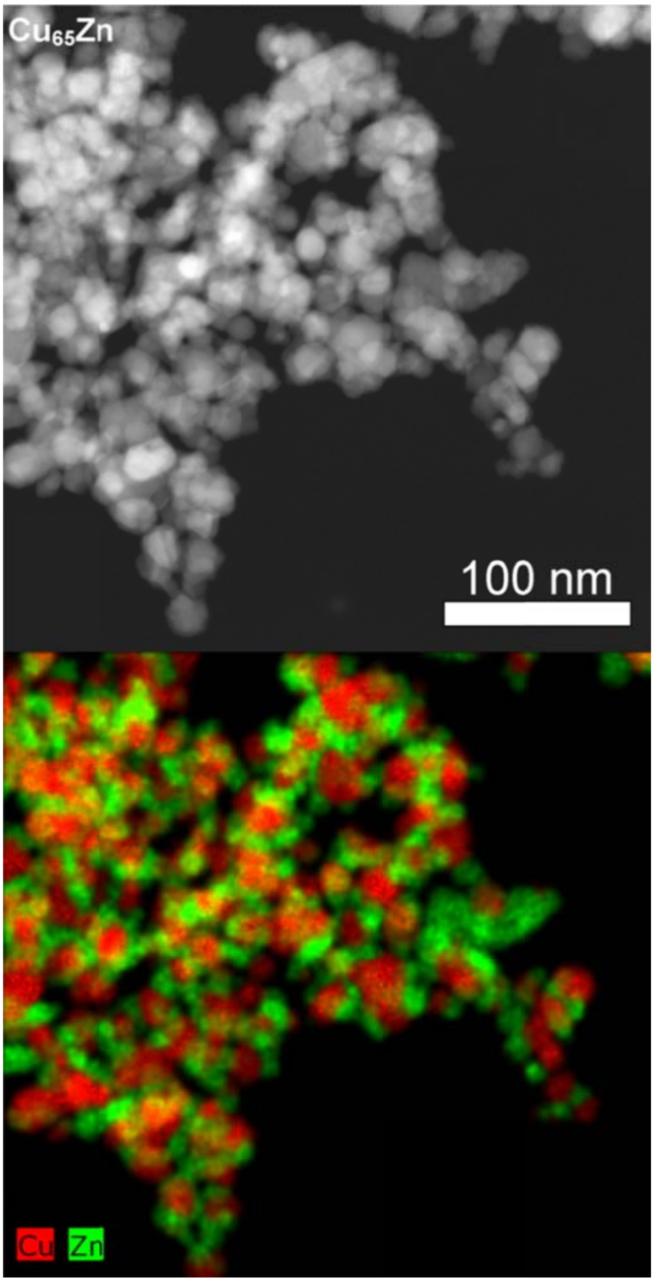

Supplementary Figure 13

STEM-HAADF image and corresponding EDX map with a pixel size of 0.36 nm of $Cu_{65}Zn$ using the TALOS F200x microscope. EDX spectra were processed using the Esprit software (Bruker AXS). Color intensities correspond to the integrated intensities of the Cu-K and Zn-K signal after background correction and are normalized per element. An automatic map filter is used. The average Cu:Zn atomic ratio in the region is 70:30, as determined by using a k-factor of 1.820 and 1.948 for Cu and Zn, respectively. The average size of the copper containing particles is 13.0 ± 2.0 nm (25 particles counted).

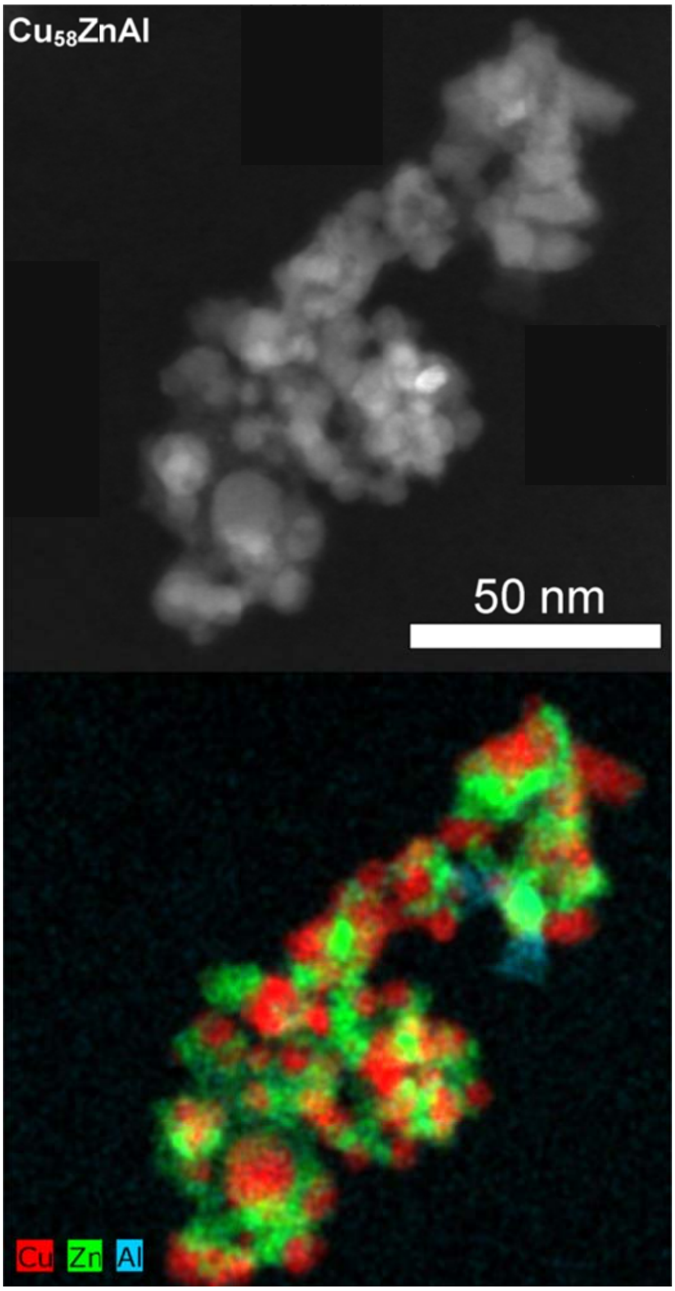

Supplementary Figure 14

STEM-HAADF image and corresponding EDX map with a pixel size of 0.12 nm of $Cu_{58}ZnAl$ using the TALOS F200x microscope. EDX spectra were processed using the Esprit software (Bruker AXS). Color intensities correspond to the integrated intensities of the Cu-K and Zn-K signal after background correction and are normalized per element. An automatic map filter is used. The average Cu:Zn:Al atomic ratio in the region is 60:31:9, as determined by using a k-factor of 1.820, 1.948 and 0.973 for Cu, Zn and Al, respectively. The average size of the copper containing particles is 6.7 ± 2.3 nm (25 particles counted).

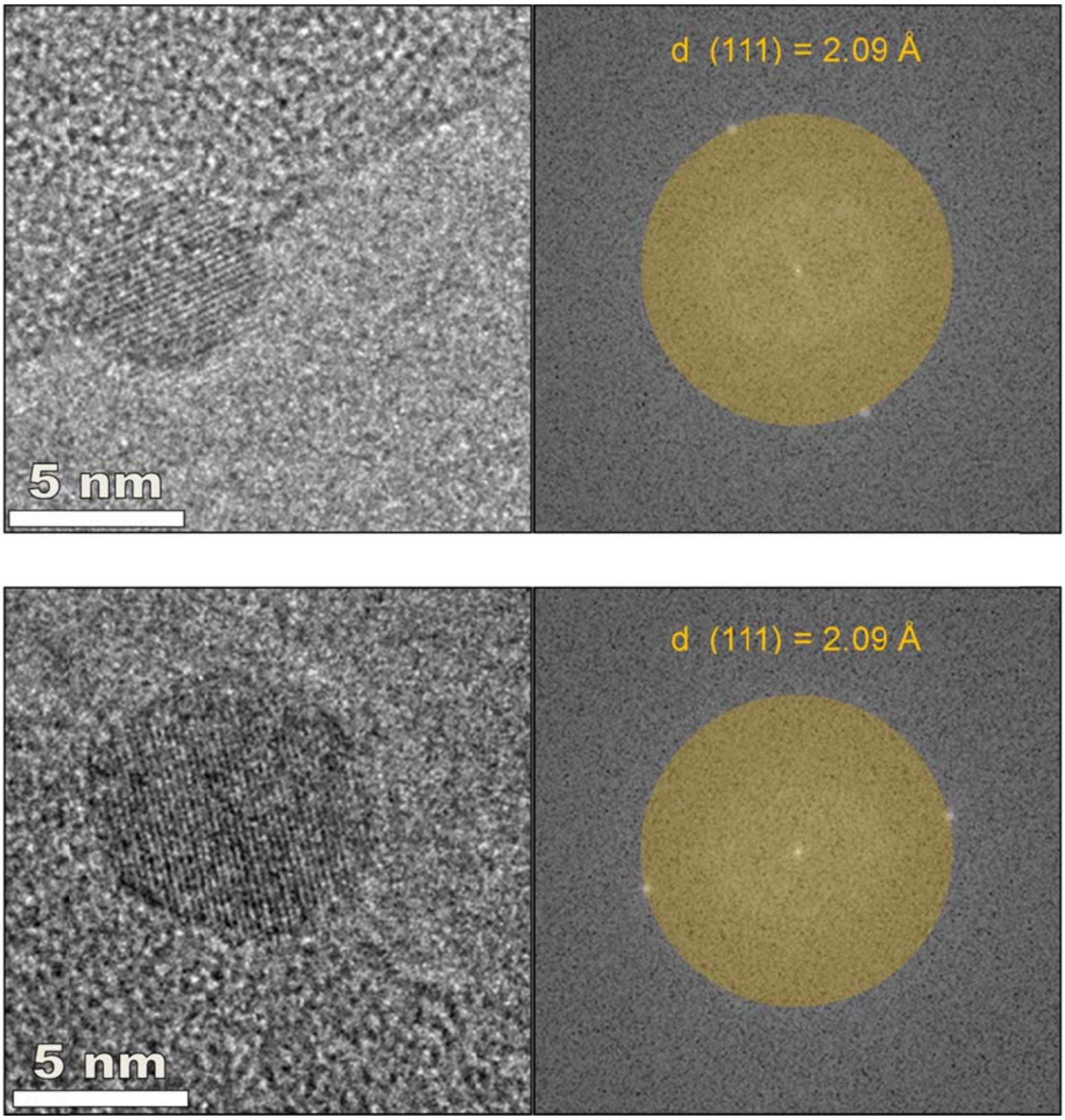

Supplementary Figure 15

High-resolution in situ TEM images of Cu_3S-NO at 1 mbar H_2 at 300 °C. The images on the right show the Fast Fourier Transforms corresponding to the TEM images on the left. The TEM images show that the copper particles are mono-crystalline. The distance between the observed lattice fringes is 2.09 Å, which corresponds to the spacing of (111) planes in metallic copper crystals.

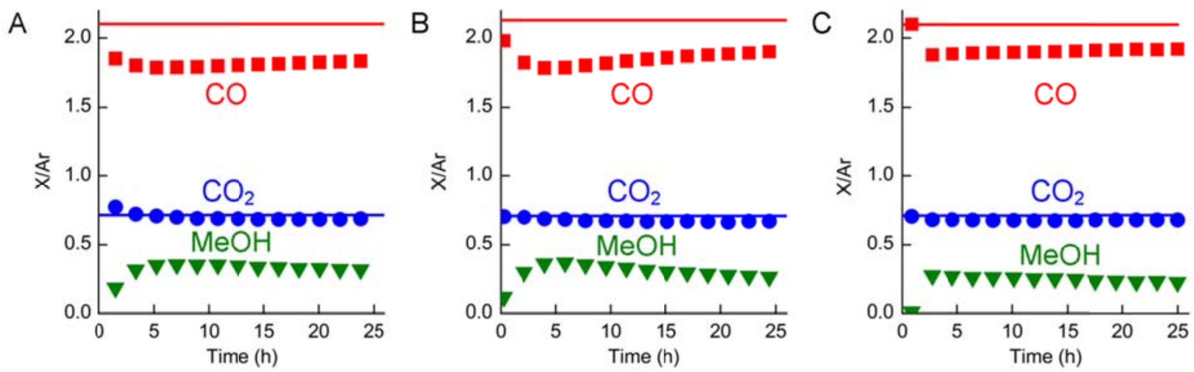

Supplementary Figure 16

Exit CO/Ar, CO_2/Ar and MeOH/Ar ratios of the integrated peak intensities in the GC chromatograms during the first 25 hours on stream for three different catalysts; A) Cu_9HSAG, B) Cu_8ZnSG(11)-NO, C) Cu_{34}Zn. Solid lines indicate the ratios in the feed composition. At t=0 the temperature is increased from 100 °C to 260 °C with a heating rate of 2 °C min^{-1}. The peak CO+CO_2 conversion after 2 to 10 h on stream is used to calculate the TOF.

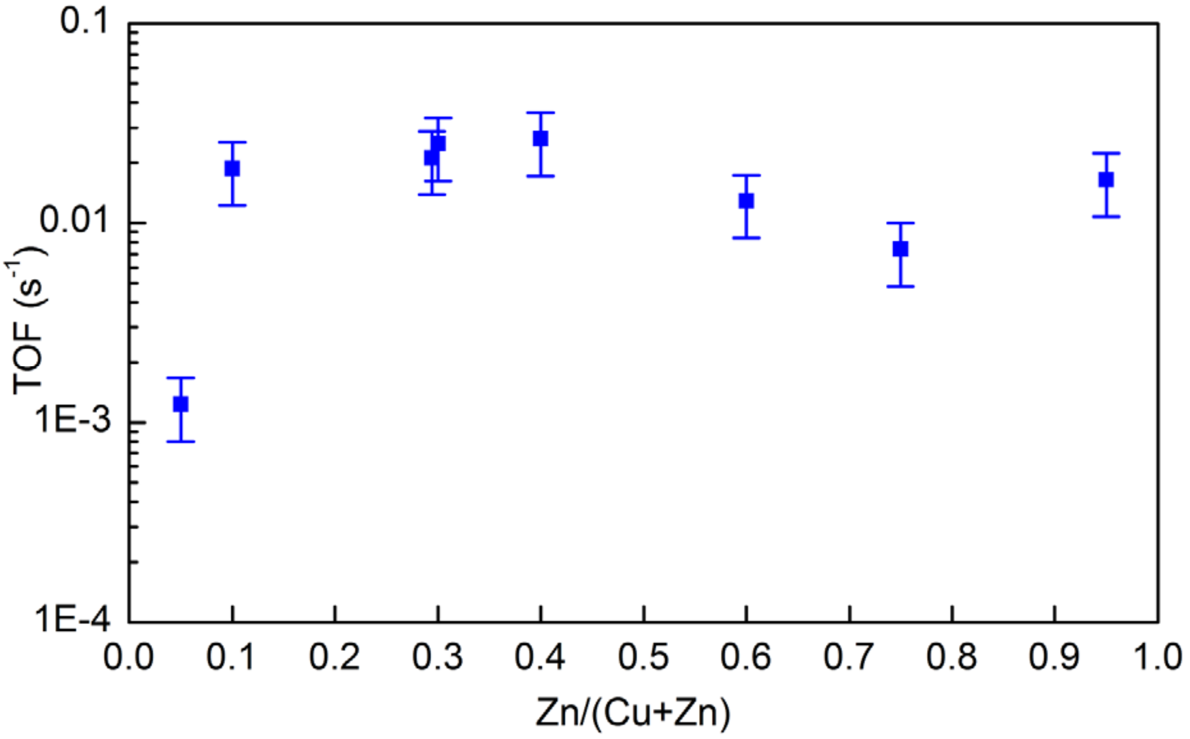

Supplementary Figure 17

Turnover frequency of the eight Cu/ZnO(/Al_2O_3) samples plotted against the zinc loading. The maximum activity was reached at Zn/(Cu+Zn) loading of about 0.1. Changes in activity at higher zinc loadings are attributed to differences in copper particle size.

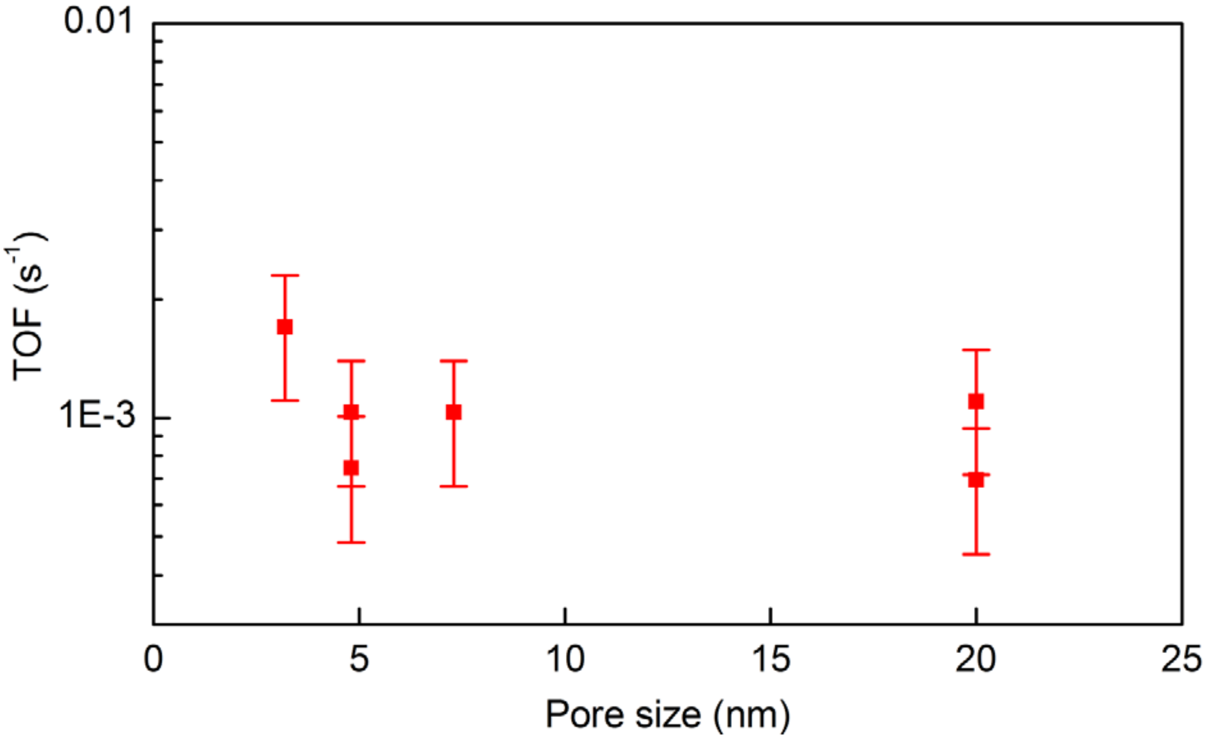

Supplementary Figure 18

Turnover frequency for methanol synthesis of the six Cu/SiO_2-N_2 samples plotted against the support pore size. Heat treatment in N_2 of non-functionalized SiO_2 impregnated with copper nitrate resulted in the formation of 2-3 nm sized copper particles, irrespective of the support porosity. Supplementary Figure 18 shows the TOFs of the six Cu/SiO_2-N_2 (red) samples plotted against the corresponding support pore size. There is no clear correlation between the support pore size and TOF indicating that the copper particles were similarly accessible for methanol synthesis irrespective of the support porosity.

Supplementary Table 1

Cu(Zn)/SiO_2 Sample list

Sample name[a]	[Cu^{2+}] (M)[b]	[Zn^{2+}] (M)[c]	Heat treatment[d]	Cu (wt%)[e]	Zn (wt%)[f]	d(Cu)$_{XRD}$ (nm)[g]	d(Cu)$_{TEM}$ (nm)[h]	CO+CO_2 conversion (%)	Activity (mol kg^{-1}_{cat} h^{-1})[i]	TOF (s^{-1})[j]
Cu_1SN-NO	0.25	-	NO	0.8[k]	-	n.d.	2.6[n]	0.6	0.14	8.9*10^{-4}
Cu_1S-N_2	0.25	-	N_2	0.9[l]	-	n.d.	2.3[n]	0.9	0.13	7.0*10^{-4}
Cu_2S-N_2	0.50	-	N_2	1.7[l]	-	n.d.	2.4[n]	2.7	0.38	1.1*10^{-3}
Cu_2S-NO	0.50	-	NO	1.9[l]	-	8.5[n]	6.5[n]	1.7	0.22	1.6*10^{-3}
Cu_3S-NO	1.00	-	NO	3.4	-	10.6[n]	6.6[n]	2.9	0.44	2.4*10^{-3}
Cu_{14}SBA16a-N_2	2.86	-	N_2	14.0	-	-	2.7[n]	13.5	4.8	1.7*10^{-3}
Cu_{15}SBA16b-N_2	3.50	-	N_2	15.0	-	-	2.4[n]	3.8	2.4	7.4*10^{-4}
Cu_7FDU(5)-N_2	2.00	-	N_2	7.1	-	-	2.8[n]	4.1	1.3	1.0*10^{-3}
Cu_5FDU(7)-N_2	1.05	-	N_2	5.1	-	-	2.6[n]	4.1	1.1	1.0*10^{-3}
Cu_8SG(3)-N_2	4.35	-	N_2	7.7[k]	-	n.d.	2.0[m]	2.2	1.3	6.6*10^{-4}
Cu_{10}SG(3)-NO	4.35	-	NO	10.0[k]	-	5.2[m], 3.2[n]	3.9[m]	5.1	1.5	1.2*10^{-3}
Cu_9SG(5)-NO	2.86	-	NO	9.1[l]	-	6.7[m], 7.3[n]	8.8[m]	7.3	1.4	2.7*10^{-3}
Cu_9SG(6)-NO	2.00	-	NO	8.6[l]	-	7.1[m], 7.6[n]	8.6[m]	12.9	1.8	3.5*10^{-3}
Cu_7SG(11)-NO	1.05	-	NO	6.7[l]	-	8.2[m], 10.2[n]	10.9[m]	3.2	0.5	1.6*10^{-3}
Cu_6SG(15)-NO	0.86	-	NO	6.1[l]	-	9.3[m], 14.6[n]	14.2[m]	3.2	0.4	2.0*10^{-3}
$Cu_{40}SiO_2$[p]	-	-	-	40.1[k]	-	5.2[m]	8.0[m]	10.5	3.9	1.5*10^{-3}
Cu_{12}ZnSBA15-N_2	2.60	1.40	N_2	11.5[k]	5.9	2.4[m]	3.3[m]	10.3	6.5	3.9*10^{-3}
Cu_{12}ZnSBA15-NO	2.60	1.40	NO	12.2[k]	5.9	3.5[m]	4.3[m]	17.3	9.1	7.2*10^{-3}
Cu_4ZnSBA16c-NO	2.60	1.40	NO	4.3[k]	2.8	n.d.	2.4[m]	7.8	3.6	4.7*10^{-3}
Cu_{10}ZnSBA16d-NO	2.60	1.40	NO	9.9[k]	6.1	7.9[n]	3.3[m]	12.1	7.9	6.5*10^{-3}
Cu_{12}ZnSBA16e-NO	2.60	1.40	NO	11.9[k]	7.2	7.3[n]	4.8[m]	16.0	10.2	7.0*10^{-3}
Cu_{11}ZnSG(5)-NO	3.25	1.75	NO	10.8[k]	5.9	2.4[n]	4.2[m]	15.8	7.2	5.2*10^{-3}
Cu_{10}ZnSG(6)-NO	2.21	1.19	NO	10.0[k]	5.3	3.2[n]	5.6[m]	14.7	9.2	1.0*10^{-2}
Cu_{12}ZnSG(6)-NO	2.60	1.40	NO	11.8[k]	6.1	4.8[n]	5.6[m]	13.1	8.3	8.1*10^{-3}
Cu_8ZnSG(11)-NO	1.30	0.70	NO	7.6[k]	4.1	5.4[n]	5.9[m]	13.1	5.4	9.5*10^{-3}
Cu_{12}ZnSG(11)-NO	2.60	1.40	NO	11.8[k]	7.3	6.4[n]	7.7[m]	12.0	7.5	1.0*10^{-2}
Cu_7ZnSG(15)-NO	1.04	0.56	NO	6.9[k]	3.9	6.8[n]	7.2[m]	5.7	2.6	5.4*10^{-3}
Cu_{16}ZnSG(15)-NO	2.60	1.40	NO	16.0[k]	8.2	9.5[n]	7.3[m]	18.0	11.5	1.3*10^{-2}
Cu_5ZnSG(25)-NO	0.65	0.35	NO	5.2[l]	2.9	6.7[n]	8.5[m]	8.3	2.2	8.1*10^{-3}
Cu_{17}ZnSG(25)-NO	2.60	1.40	NO	17.0	9.4	9.9[n]	10.1[m]	12.9	12.4	1.6*10^{-2}

[a]Cu_x stands for the copper weight loading, Zn indicates the presence of zinc (in a 65/35 Cu/Zn molar ratio), and the metal contents are followed by the abbreviation of the support. "N_2" or "NO" indicates the gas atmosphere at which the sample is heat treated at 350 °C after incipient wetness impregnation and drying, i.e. with either 750 ml min^{-1} N_2 (N_2) or 375 ml min^{-1} 2% NO/N_2 (NO). [b]Cu^{2+} is the copper ion concentration in the aqueous impregnation solution, [c]Zn^{2+} is the zinc ion concentration in the aqueous impregnation solution. [d]Heat treatment at 350 °C of sample after incipient wetness impregnation with either 750 ml min^{-1} N_2 (N_2) or 375 ml min^{-1} 2% NO/N_2 (NO). [e]Nominal copper loading in the final catalyst (assuming copper is metallic and zinc oxidic). [f]Nominal zinc loading in the final catalyst. [g]Cu or CuO crystallite size as determined by XRD with the Scherrer equation. [h]Number-averaged particle size as determined with (S)TEM. [i]Peak methanol productivity after 2 to 10 h on stream normalized per kg of catalyst per hour. [j] Turnover Frequency for methanol synthesis normalized per surface copper atom after 2 to 10 h on stream. [k]Copper loading determined with TPR-TCD. [l]Copper loading determined with TPR-MS. [m]Cu crystal or particle size. [n]CuO crystal or particle size. [p]Cu/SiO_2 prepared via precipitation followed by reduction.

Supplementary Table 2

Cu(Zn)/C sample list

Sample name[a]	$[Cu^{2+}]$[b]	$[Zn^{2+}]$[c]	Cu (wt%)[d]	Zn (wt%)[e]	$d(Cu))_{XRD}$ (nm)[f]	$d(Cu)_{TEM}$ (nm)[g]	$CO+CO_2$ conversion (%)	Activity (mol kg^{-1}_{cat} h^{-1})[h]	TOF (s^{-1})[i]
Cu_9HSAG	2.00	-	8.6	-	3.9	5.4	12.1	2.5	$3.5*10^{-3}$
$Cu_9Zn_{0.2}$HSAG	2.00	0.04	8.6	0.2	3.2	6.3	7.1	7.1	$1.1*10^{-2}$
$Cu_9Zn_{0.5}$HSAG	2.00	0.11	8.5	0.5	3.3	8.0	11.4	13.7	$3.1*10^{-2}$
$Cu_9Zn_{0.7}$HSAG	2.00	0.15	8.6	0.7	4.9	5.7	9.6	20.3	$3.1*10^{-2}$
$Cu_9Zn_{1.0}$HSAG	2.00	0.22	8.5	1.0	3.2	8.9	8.4	7.7	$1.8*10^{-2}$
$Cu_8Zn_{2.2}$HSAG	2.00	0.50	8.4	2.2	10.2	8.7	7.9	7.5	$1.8*10^{-2}$
$Cu_8Zn_{4.5}$HSAG	2.00	1.08	8.1	4.5	4.0	9.3	5.4	8.8	$2.5*10^{-2}$
$Cu_8Zn_{8.0}$HSAG	2.00	2.00	7.7	8.0	3.5	8.7	10.3	9.6	$2.3*10^{-2}$
CuZnCX(4)	1.95	1.05	4.4	2.4	11.6	5.4	7.2	5.1	$1.3*10^{-2}$
CuZnCX(5)	1.32	0.71	4.4	2.4	9.9	7.3	7.0	6.5	$2.6*10^{-2}$
CuZnCX(8)	0.75	0.40	3.9	2.1	7.1	7.6	5.1	4.8	$2.1*10^{-2}$
CuZnCX(12)	0.75	0.40	4.8	2.7	8.1	10.6	12.3	6.6	$3.6*10^{-2}$

[a]Cu_x stands for the copper weight loading, Zn_x stands for the zinc weight loading, and the metal contents are followed by the abbreviation of the support. [b]Cu^{2+} is the copper ion concentration in the aqueous impregnation solution. [c]Zn^{2+} is the zinc ion concentration in the aqueous impregnation solution. [d]Nominal copper loading in the final catalyst (assuming copper is metallic and zinc oxidic). [e]Nominal zinc loading in the final catalyst. [f]Cu crystallite size as determined by XRD with the Scherrer equation. [g]Number-averaged particle size as determined with TEM. [h]Peak methanol productivity after 2 to 10 h on stream normalized per kg of catalyst per hour. [i]Turnover Frequency for methanol synthesis normalized per surface copper atom after 2 to 10 h on stream.

Supplementary Table 3

Cu/ZnO(/Al_2O_3) sample list

Catalyst[a]	$[Cu^{2+}]$[b]	$[Zn^{2+}]$[c]	$[Al^{3+}]$[d]	Cu (wt%)[e]	ZnO (wt%)[f]	Al_2O_3 (wt%)[g]	$d(ZnO)_{xrd}$ (nm)[h]	$d(Cu)_{xrd}$ (nm)[i]	$d(Cu)_{TEM}$ (nm)[j]	$CO+CO_2$ conversion (%)	Activity (mol kg^{-1} h^{-1})[k]	TOF (s^{-1})[l]
Cu_4Zn	0.1	1.9	-	4.2	95.8	-	11.8	7.9	6.6	11.3	4.4	$1.7*10^{-2}$
$Cu_{21}Zn$	0.5	1.5	-	20.7	79.3	-	12.3	5.9	5.2	21.1	13.3	$7.4*10^{-3}$
$Cu_{34}Zn$	0.8	1.2	-	34.2	65.8	-	12.9	5.5	6.9	9.0	30.1	$1.3*10^{-2}$
$Cu_{54}Zn$	1.2	0.8	-	53.9	46.1	-	12.3	11.9	11.8	11.7	46.9	$2.7*10^{-2}$
$Cu_{65}Zn$	1.4	0.6	-	64.6	35.4	-	13.9	13.5	13.6	11.7	55.2	$2.5*10^{-2}$
$Cu_{88}Zn$	1.8	0.2	-	87.5	12.5	-	14.3	24.4	30.4	9.4	21.6	$1.9*10^{-2}$
$Cu_{94}Zn$	1.9	0.1	-	93.7	6.3	-	16.3	31.6	33.7	0.3	0.79	$1.2*10^{-3}$
$Cu_{58}ZnAl$	1.2	0.5	0.3	57.7	30.8	11.6	8.9	8.2	7.2	17.8	80.7	$2.1*10^{-2}$

[a]Cu_x stands for the copper weight loading, Zn indicates the presence of zinc oxide, Al indicates the presence of alumina. [b]Cu^{2+} is the copper ion concentration in the aqueous impregnation solution. [c]Zn^{2+} is the zinc ion concentration in the aqueous impregnation solution. [d]Al^{3+} is the aluminum ion concentration in the aqueous impregnation solution. [e]Nominal copper loading in the final catalyst (assuming catalyst compositions of metallic copper, zinc oxide and alumina. [f]Nominal zinc oxide loading in the final catalyst. [g]Nominal alumina loading in the final catalyst. [h]ZnO crystallite size as determined by XRD with the Scherrer equation. [i]Cu crystallite size as determined by XRD with the Scherrer equation. [j]Number-averaged particle size as determined with TEM, [k]Peak methanol productivity after 2 to 10 h on stream normalized per kg of catalyst per hour. [l]Turnover Frequency for methanol synthesis normalized per surface copper atom after 2 to 10 h on stream.

Supplementary Table 4

Data derived from N_2-physisorption isotherms on the silica supports shown in Supplementary Figure 1; Pore Volume (PV) determined at p/p_0=0.9975; average pore size from BJH analysis (4V/A) of the adsorption isotherm.

Support	BET (m^2g^{-1})	PV (cm^3g^{-1})	Average pore size (nm)
SG(3)	720	0.52	3.4
SG(5)	520	0.82	4.5
SG(6)	575	0.63	6.1
SG(11)	355	1.03	10.6
SG(15)	330	1.20	15.0
SG(25)	300	1.43	24.8
S	100	0.58	20.3
SN	75	0.53	23.0
SBA-15	775	1.00	4.3
$Cu_{40}SiO_2$	375	0.59	5.6
FDU(5)	815	0.66	4.8
FDU(7)	390	0.90	7.3
SBA-16a	1255	0.86	3.2
SBA-16b	885	0.98	4.8
SBA-16c	570	0.34	3.0
SBA-16d	925	0.82	4.3
SBA-16e	930	1.00	4.5

Supplementary Table 5

Data derived from N_2-physisorption isotherms on the carbon supports shown in Supplementary Figure 2; Pore Volume (PV) determined at p/p_0=0.9975; average pore size from BJH analysis (4V/A) of the adsorption isotherm.

Support	BET (m^2g^{-1})	PV (cm^3g^{-1})	Average pore size (nm)
HSAG	592	0.79	5.5
CX(4)	637	0.39	4.1
CX(5)	491	0.47	4.6
CX(8)	655	0.86	8.4
CX(12)	639	1.15	12.0

Supplementary Table 6

Data derived from N_2-physisorption isotherms on the precipitated Cu/ZnO(/Al_2O_3) samples shown in Supplementary Figure 3; Pore Volume (PV) determined at p/p_0=0.9975; average pore size from BJH analysis (4V/A) of the adsorption isotherm.

Sample	BET (m^2g^{-1})	PV (cm^3g^{-1})	Average pore size (nm)
Cu_4Zn	42	0.29	28.0
$Cu_{21}Zn$	37	0.31	37.0
$Cu_{34}Zn$	36	0.30	33.9
$Cu_{54}Zn$	28	0.21	30.3
$Cu_{58}ZnAl$	77	0.28	12.7
$Cu_{65}Zn$	47	0.35	27.9
$Cu_{88}Zn$	14	0.12	37.4
$Cu_{94}Zn$	9	0.06	28.8

Supplementary Methods

Supports

Silica gels (SG) were obtained from Merck (SG(5)) and Grace-Davison (SG(3), SG(6), SG(11), SG(15), SG(25)). The numbers in the brackets indicate the average pore size as determined from by BJH analysis (4V/A) of the adsorption branch of the N_2 physisorption isotherms.

High surface area graphite 500 (HSAG) was obtained from Timcal Ltd., Switzerland.

Stöber silica (S) and **functionalized Stöber silica** (SN) were synthesized as described in Van den Berg et al.[1]

SBA15 was synthesized as described in Prieto et al.[2]

SBA16 silica mesostructures were synthesized with varying porosity. Block-copolymers Pluronic F127 ($EO_{106}PO_{70}EO_{106}$) and Pluronic P123 ($EO_{20}PO_{70}EO_{20}$) from Sigma-Aldrich, 1-butanol (p.a., Acros), and tetraethyl ortosilicate (TEOS, 99%, Sigma-Aldrich) were used as received. SBA16c was synthesized following the procedure reported by Kim et al.[3] using a synthesis gel with the following molar composition: 0.0016 P123 / 0.0037 F127 / 1.0 TEOS / 4.4 HCl / 140 H_2O. The copolymers were dissolved at room temperature in HCl/H_2O. TEOS was subsequently added dropwise while using a magnetic stirrer and the gel was aged in an oven at 35 ºC for 20 h under static conditions. The mixture was hydrothermally treated at 60 ºC for 24 h. SBA16a, SBA16b, SBA16d and SBA16e were prepared using 1-butanol (BuOH) as a swelling agent at low acid concentrations as described by Kleitz et al.[4] A synthesis gel with the following molar ratios was used: 0.0035 F127 / 1.79 BuOH / 1.0 TEOS / 0.91 HCl / 120 H_2O. After the block-copolymer was dissolved in HCl/H_2O, 1-butanol was added and the mixture was stirred at 40 °C for ½ h (SBA16a and SBA16b) or 1 h (SBA16d and SBA16e). Then, TEOS was added dropwise under stirring and the gel was aged in an oven at 40 °C for 20 h under static conditions. SBA16b was stirred for 5 min prior to the aging. The mixtures were further treated for 48 h at 90 ºC (SBA16b and SBA16d), 72 h at 100 ºC (SBA16a) or 24 h at 120 ºC (SBA16e). In all cases, after the hydrothermal treatment, the resulting solids were filtered, extensively washed with deionized water, and dried at 60 ºC (SBA16a and SBA16b) or 120 ºC for 10 h (SBA16d and SBA16e). Finally, the products were calcined at 350 ºC (SBA16b), 500 ºC (SBA16a) or 540 ºC (SBA16c, SBA16d and SBA16e) in a muffle oven to remove the copolymer template.

FDU12 silica materials were synthesized employing 1,3,5-trimethylbenzene (TMB) as a micelle swelling agent and tetraethyl ortosilicate (TEOS) as the silica source. Pluronic F127 (Sigma-Aldrich) and KCl (p.a., Acros) were dissolved in HCl/H_2O at room temperature. TMB (99%, Acros) was subsequently added and the mixture stirred in a polyethylene bottle at 14 ºC for 24 hours. Then TEOS (99%, Sigma-Aldrich) was added drop-wise under stirring and the mixture further stirred at 14 ºC for 20 hours. The final synthesis gel molar composition was: 0.004 F127 :

1.7 KCl : 0.93 TMB : 1.0 TEOS : 6.1 HCl : 157 H_2O. Subsequently, the mixture was divided into two aliquots, transferred to Teflon-lined autoclaves and treated hydrothermally for 48 hours in an oven. The temperature of this hydrothermal treatment was 60 ºC (FDU(5)) and 130 ºC (FDU(7)). The resulting solids were recovered by filtration and dried at 60 ºC for 10 hours. Next, an additional hydrothermal treatment was carried out in order to remove the Na residues that might remain in the silica product. The silica solid was re-dispersed in a 2M HCl aqueous solution (70 ml g^{-1} solid) and the resulting suspension was transferred to a polypropylene bottle and hydrothermally treated at 70 ºC for 72 hours. Next, the solid was recovered by filtration, washed extensively with 2M HCl and then water until the pH of the washing waters was 6. The solid was dried at 120 ºC for 10 hours and then calcined in a muffle oven at 540 ºC for 5 hours.

Carbon Xerogels (CX) were synthesized through resorcinol condensation catalyzed by sodium carbonate followed by pyrolysis. An amount of 12.89 g formaldehyde (37%, 12% methanol, Fisher chemical) and 8.65 g of resorcinol (99%, Sigma Aldrich) were successively added to 0.017g sodium carbonate (>99%, Acros Chemicals) dissolved in 3.08 g (CX4), 5.14 g (CX5), 8.55 (CX8) or 12.00 g (CX12) water. The mixtures were kept at room temperature for one day, heated to 60 °C for one day and heated to 90 °C for three days. The solid materials were crushed (< 3 mm) and washed twice with acetone for 1 h followed by drying at room temperature for 1 h and one time with acetone for 8 h followed by drying at room temperature for 72 h. Subsequently, the carbon xerogels were pyrolysed in a tubular oven in a 100 ml min^{-1} argon flow at 800 °C (5 °C min^{-1}) for 10 h and reduced at 600 °C (10 °C min^{-1}) for 5h in a100 ml min^{-1} flow of 50% H_2/Ar.

Samples

Cu(Zn)/SiO$_2$. Copper(/Zinc) catalyst were prepared using different silica supports, including silica gels, (aminopropyl-functionalized) Stöber silica, SBA15, SBA16, FDU; see supporting information for synthesis details and N_2-physisorption data. The supports were first dried at 150 °C under vacuum for 1 h to remove adsorbed water. Thereafter, the supports were incipient wetness impregnated with an aqueous solution of 0.25 to 4.35 M copper nitrate ($Cu(NO_3)_2$•3 H_2O, Acros Organics) or copper and zinc nitrate with a total metal concentration between 1.0 and 5.0 M ($Zn(NO_3)_2$•6H_2O, Sigma Aldrich) in a 65/35 atomic Cu/Zn ratio in 0.1 M HNO_3. The samples (~2 g) were subsequently dried under vacuum at room temperature overnight and heat treated at 350 °C (2 °C min^{-1}) in a plug-flow reactor with either a flow of 750 ml min^{-1} N_2 or 375 ml min^{-1} 2% NO/N_2. The nominal copper loading in the samples heat treated in N_2 was kept below 2 Cu atoms per nm^2 of support to avoid the formation of copper particles larger than 5 nm.[1,5] Cu/SiO$_2$ was also prepared via homogeneous deposition precipitation of copper on colloidal silica followed by a hydrothermal treatment and reduction as described by van den Berg et al.[6] Part of the as-prepared samples was used for catalysis following *in-situ* reduction as described below, and part (~1 g) was used for characterization. Characterization was done on as-prepared samples and on samples after reduction and passivation, mimicking the activation procedure used prior to catalysis. The reduction was performed in a flow of 100 ml min^{-1} of 20% H_2 in Ar at 250 °C (2 °C min^{-1}) for 2½ h. After reduction, samples were passivated for 15 min by slowly exposing them at room temperature to air diluted with N_2. The samples were stored in a glove box under argon atmosphere. Supplementary Table 1 shows a list of the samples, synthesis details, their main physicochemical characteristics (notably metal loading and dispersion) and at which catalyst synthesis stage these characteristics were determined, i.e. at the oxidized or reduced state.

Cu(Zn)/C. Copper/(Zinc) catalyst were prepared using different carbon supports, including carbon xerogels and high surface area graphite (HSAG); see supporting information for synthesis details and N_2-physisorption data. The supports were first dried at 150 °C under vacuum for 1 h to remove absorbed water. Thereafter, the carbon xerogels were incipient wetness impregnated with an aqueous solution of copper and zinc nitrate in a 65/35 atomic Cu/Zn ratio in 0.1 M HNO_3 with a total metal concentration between 1.15 and 3.0 M, and HSAG was incipient wetness co-impregnated with aqueous solutions of 2 M copper nitrate and 0 to 2 M zinc nitrate in 0.1 M HNO_3. The samples (~2 g) were subsequently dried under vacuum at room temperature overnight and reduced at 230 °C (2 °C min^{-1}) in a plug-flow reactor with 100 ml min^{-1} 20% H_2/N_2. After the reduction treatment the samples were passivated for 15 minutes by slowly exposing the sample to diluted air/N_2 at room temperature. The samples were subsequently characterized. Furthermore, the samples were loaded in a reactor, rereduced, and tested for their

performance in the methanol synthesis reaction. Supplementary Table 2 shows a list of the samples and their physicochemical characteristics.

Cu/ZnO(/Al_2O_3). Cu/ZnO(/Al_2O_3) catalysts were prepared by co-precipitation. Aqueous solutions of Cu, Zn and Al nitrates in varying atomic ratios with a total metal concentration of 2 M were added simultaneously with a 1.6 M Na_2CO_3 solution to deionized water at 65 °C at a controlled rate of 20 ml min^{-1} in order to keep the pH constant around 8. The obtained precipitates were aged for 1 h at 70 °C in the mother liquor under stirring. The samples were extensively washed with water and dried at 80 °C. The dried filter cake was crushed and sieved to 0.3 – 0.6 mm and calcined in a muffle oven at 325 °C. The samples were subsequently pressed, pelletized, crushed and sieved to obtain the right sieve fraction for catalytic testing. For the pelletization of the samples 2 to 4% graphite was added as a lubricant. Part of the as-prepared samples was used for catalysis following *in-situ* reduction as described below, and part (~1 g), intended for characterization, was reduced in a flow of 100 ml min^{-1} of 20% H_2 in Ar at 250 °C (2 °C min^{-1}) for 2½ h. After reduction the samples were passivated for 15 min by slowly exposing the samples at room temperature to air diluted with N_2. The samples were stored in a glove box under argon atmosphere and subsequently characterized. Supplementary Table 3 shows a list of the prepared samples and their physicochemical characteristics.

Characterization

TPR. Temperature programmed reduction (TPR) measurements of the Cu(Zn)/SiO_2 samples after heat treatment in N_2 or 2% NO/N_2 were either done with a Micromeritics Autochem II ASAP 2920 (TPR-TCD) or in a fixed-bed flow setup with online gas analysis performed by a quadrupole mass spectrometer (Balzers GAM 445, TPR-MS). Prior to TPR-TCD measurements, the samples were dried at 80 °C for 1 h (CuZn/SiO_2 samples) or at 120 °C for 0.5 h (Cu/SiO_2 samples). Subsequently the temperature was increased to 500 °C (10 °C min^{-1}, CuZn/SiO_2 samples) or 300 °C (5 °C min^{-1}, Cu/SiO_2 samples) under a flow of 5% H_2/Ar. During this treatment the H_2 concentration was determined with a thermal conductivity detector (TCD). TPR-MS measurements were performed by reduction at 220 °C (2 °C min^{-1}) in a 100 ml min^{-1} flow of 1% H_2/He for 7 hours. The copper loading was estimated from the H_2 consumption by assuming the reduction stoichiometry: $CuO + H_2 \rightarrow Cu + H_2O$.

N_2-physisorption. N_2 physisorption measurements on the supports were performed at - 196 °C using a Micromeritics Tristar 3000 V6.08 apparatus. Prior to the measurements, the samples were outgassed at 130 °C in a nitrogen flow for 14 h. The Brunauer-Emmet-Teller (BET) method was used to calculate the specific surface areas.[7] The pore volumes were determined at p/p_0 = 0.9975. Barrett-Joyner-Halenda (BJH) method analysis (4V/A) of the adsorption branch was used to obtain the average pore size.[8] The N_2-physisorption data of all the supports and precipitated catalysts after reduction and passivation are shown below.

XRD. For impregnated Cu(Zn)/SiO_2 samples, diffractograms were taken after heat treatment in N_2 or 2% NO/N_2. For precipitated Cu/ZnO(/Al_2O_3), impregnated Cu(Zn)/C and some of the Cu/SiO_2 samples, diffractograms were (also) taken after reduction and passivation. For these reduced and passivated catalysts, the specimen holder was loaded in the glove box and subsequently sealed. Diffractograms were collected at room temperature from 20° to 70° (2θ). No background correction or smoothening was applied. Copper and zinc oxide crystallite sizes were estimated by applying the Scherrer equation to the (111) diffraction of Cu (2θ = 50.5°, k = 0.9) or the (-111/002) diffraction of CuO (2θ = 41.5°, k = 0.9), and the (100) diffraction of ZnO (2θ = 37.0°, k = 0.9), respectively.[9]

(S)TEM. TEM micrographs and corresponding particle size distributions are shown in Figure 1 and Supplementary Figures 5, 6 and 7. CuZn/SG and CuZn/SBA16 samples were imaged in HAADF-STEM mode with a Tecnai 20FEG microscope (FEI). Cu/SBA16, Cu/FDU and Cu/SG(3) samples were imaged in HAADF-STEM mode with a TALOS F200x microscope. All other samples were imaged in bright-field mode with a Tecnai 12 electron microscope (FEI). The images A and C in Figure 1, Figure 2 and Supplementary Figure 15 were acquired using an image-aberration corrected Titan 80-300 ETEM (FEI). Prior to an experiment, the image aberration corrector was tuned using a cross-grating (Agar S106) and the spherical aberration

coefficient was set in the range of −10 to −20 μm. TEM samples of Cu_2S-N_2, Cu_3S-NO and $Cu_{40}SiO_2$ were prepared by grinding and dispersing the resulting powder on stainless steel grids. The samples were inserted in the microscope using a Gatan heating holder (model 628). Cu_2S-N_2 was reduced at 350 °C at 1 mbar H_2 for 30 min and TEM images were subsequently acquired at these conditions with an electron dose-rate of 20 electrons per $Å^2$ per second) (Figure 1A).Cu_3S-NO was reduced at 300 °C at 1 mbar H_2 for 30 min and TEM images were subsequently acquired at these conditions with an electron dose-rate of 100 electrons per $Å^2$ per second. Reduced and passivated $Cu_{40}SiO_2$ was re-reduced in the electron microscope at 250 °C at 1 mbar H_2 for 45 min and TEM images were subsequently acquired at these conditions with an electron dose-rate of 10 (Figure 1C) or 100 electrons per $Å^2$ per second (Figure 2).

Cu/S, Cu/SN, Cu/SBA16 and Cu/FDU samples are imaged after heat treatment in N_2 or 2% NO/N_2. All the other samples are imaged after reduction and passivation.

The Cu/SG(3), Cu(Zn)/C, Cu/ZnO(/Al_2O_3) after reduction and passivation and Cu/(functionalized)Stöber silica, Cu/FDU and Cu/SBA16 after heat treatment in N_2 or 2% NO/N_2 were prepared by grinding followed by sonication in ethanol. A droplet of the resulting ethanol suspensions was deposited on carbon coated copper TEM grids (Agar S162 200 Mesh Cu). Other Cu(Zn)/SiO_2 samples after reduction and passivation were ground, embedded in a two-component epoxy resin (Epofix, EMS), cured at 60 °C overnight, and cut into thin sections (50-100 nm) using a Diatome Ultra 35° diamond knife mounted on an Ultracut E microtome (Reichert-Jung). Sections were deposited on a copper TEM grid.

In the case of CuZn/SiO_2 and CuZn/C samples crystalline zinc species were not detected prior to catalysis by XRD (Figure 3 and Supplementary Figure 4). Consistent with this finding, STEM-EDX did not reflect compact particles of ZnO. Particles of higher contrast and more spherical shapes were identified to consist mainly of copper (Supplementary Figures 9 to 11). The size of high-contrast spherical particles in (S)TEM was therefore used to determine the copper particle size distribution.

In the case of Cu/ZnO/(Al_2O_3) samples XRD showed the presence of ZnO crystallites with an average size varying from 9 to 16, and Cu crystallites with an average size varying from 6 to 32 (Supplementary Table 3 and Supplementary Figure 4). STEM-EDX images showed that copper and zinc oxide particles were well mixed, that zinc oxide particles had a rather similar size of about 10 to 15 nm irrespective of the composition in line with XRD, and that (different sized) higher contrast and more spherically shaped particles consisted mainly of copper while lower contrast and in some cases more anisotropically shaped particles consisted mainly of ZnO (Supplementary Figures 12 to 14). For the different samples the estimated ratio between higher contrast and more spherical particles and lower contrast and more anisotropic particles seemed to correspond to the Cu/ZnO weight ratio. The projected area of the higher contrast and spherical particles in TEM was therefore considered to reflect the copper particle size distribution. With this analysis, it cannot be ruled out that zinc oxide particles were fully excluded from the as-

determined distributions. However, the number-average particle sizes of the distributions were similar to the average copper crystallite sizes determined with XRD and the average copper particle sizes in the STEM-EDX images, evidencing the validity of the criteria used to identify the copper particles in the TEM images.

Catalytic activity

The methanol productivity is based upon the CO + CO_2 conversion. The conversion of CO or CO_2 is calculated by the difference in CO/Ar or CO_2/Ar ratio between chromatograms taken during reaction and chromatograms taken of the gas feed before reaction (Supplementary Equations 1 and 2).

$$X_{CO} = \frac{\frac{CO_{feed}}{Ar_{feed}} - \frac{CO_{reaction}}{Ar_{reaction}}}{\frac{CO_{feed}}{Ar_{feed}}} \quad 1$$

$$X_{CO2} = \frac{\frac{CO2_{feed}}{Ar_{feed}} - \frac{CO2_{reaction}}{Ar_{reaction}}}{\frac{CO2_{feed}}{Ar_{feed}}} \quad 2$$

X_{CO} and X_{CO2} are the conversion of CO and CO_2, respectively. CO_{feed}, $CO2_{feed}$ and Ar_{feed} are the peak areas of the corresponding gases in the TCD chromatograms of the syngas feed before catalysis. $CO_{reaction}$, $CO2_{reaction}$ and $Ar_{reaction}$ are the peak areas of the exit gas composition during catalysis. The methanol productivity is calculated by the CO + CO_2 conversion (Supplementary Equation 3).

$$n_{MeoH} = X_{CO} * n_{CO} + X_{CO2} * n_{CO2} \quad 3$$

n_{MeOH} is the rate of methanol production. n_{CO} or n_{CO2} is the molar flow rate of CO or CO_2, respectively, into the reactor, calculated via Supplementary Equation 4 and 5.

$$n_{CO} = \frac{Q_{CO} * P_{ref}}{RT_{ref}} * \varphi \quad 4$$

$$n_{CO2} = \frac{Q_{CO2} * P_{ref}}{RT_{ref}} * \varphi \quad 5$$

Q_{CO} or Q_{CO2} are the molar fractions of CO and CO_2 in the syngas feed, P_{ref} is the pressure and T_{ref} the temperature at which the mass flow controller has been calibrated and R is the gas constant. φ is the syngas flow into the reactor as measured by the mass flow controller. To obtain catalyst mass-based productivities (P) The calculated rate of methanol production was divided by the mass of the catalyst (m_{cat}) according to Supplementary Equation 6. The catalyst mass is corrected for the loss of mass during reduction prior to catalysis.

$$P = \frac{n_{MeOH}}{m_{cat}} \quad 6$$

Turnover frequencies (TOF) were calculated per surface metal atom. The number of surface atoms was based on the TEM copper particle size distributions assuming fully accessible spherical particles. First, the surface averaged particle size (PS) was calculated via Supplementary Equation 7.

$$PS = \frac{\sum_1^n D_i^3}{\sum_1^n D_i^2} \qquad 7$$

D_i is the diameter of the ith particle. The dispersion (ratio between copper surface atoms and total copper atoms) was calculated according to Supplementary Equation 8.

$$dispersion = \frac{6V_m}{A_m * PS} \qquad 8$$

V_m is the molar volume and A_m the molar area of the particles. In the case of copper V_m is 7.09 $*10^{21}$ nm^3 and A_m is $4.10*10^{22}$ nm^2. The dispersion of copper ($dispersion_{cu}$) is therefore given by Supplementary Equation 9 with PS in nm.

$$dispersion_{cu} = \frac{1.04}{PS} \qquad 9$$

The molar amount of copper surface atoms in the catalyst (Cu_{surf}) was calculated by Supplementary Equation 10

$$Cu_{surf} = dispersion_{cu} * \frac{Wt_{cu}}{M_{cu}} * m_{cat} \qquad 10$$

Where Wt_{cu} is the weight fraction of copper in the catalyst and M_{cu} the molar mass of copper. TOFs were calculated according to Supplementary Equation 11

$$TOF = \frac{n_{MeOH}}{Cu_{surf}} \qquad 11$$

Supplementary References

1 van den Berg, R. *et al.* Support functionalization to retard Ostwald ripening in copper methanol synthesis catalysts. *ACS Catal.* **5**, 4439-4448 (2015).

2 Prieto, G., Zečević, J., Friedrich, H., de Jong, K. P. & de Jongh, P. E. Towards stable catalysts by controlling collective properties of supported metal nanoparticles. *Nat. Mater.* **12**, 34-39 (2013).

3 Kim, T.-W. *et. al.* Tailoring the pore structure of SBA-16 silica molecular sieve through the use of copolymer blends and control of synthesis temperature and time. *J. Phys. Chem. B* **108,** 11480-11489 (2004).

4 Kleitz, F., Solovyov, L. A., Anilkumar, G. M., Choi, S. H., Ryoo, R. Transformation of highly ordered large pore silica mesophases (*Fm3m, Im3m* and *p6mm*) in a ternary triblock copolymer-butanol-water system *Chem. Commun.* **10**, 1536-1537 (2004).

5 Prieto, G., Meeldijk, J. D., de Jong, K. P. & de Jongh, P. E. Interplay between pore size and nanoparticle spatial distribution: Consequences for the stability of $CuZn/SiO_2$ methanol synthesis catalysts. *J. Catal.* **303**, 31-40 (2013).

6 van den Berg, R. *et al.* Impact of the synthesis route of supported copper catalysts on the performance in the methanol synthesis reaction. *Catal. Today*, **272**, 87-93 (2015).

7 Brunauer, S., Emmett, P. H. & Teller, E. Adsorption of gases in multimolecular layers. *J. Am. Chem. Soc.* **60**, 309-319 (1938).

8 Barret, E. P., Joyner, L. G. & Halenda, P. P. The determination of pore volume and area distributions in porous substances. I. computations from nitrogen isotherms. *J. Am. Chem. Soc.* **73**, 373-380 (1951).

9 Patterson, A. L. The Scherrer formula for X-ray particle size determination. *Phys. Rev.* **56**, 978-982 (1939).
